# Accuracy of HPV E6/E7 oncoprotein tests to detect high-grade cervical lesions: a systematic literature review and meta-analysis

**DOI:** 10.1038/s41416-023-02490-w

**Published:** 2023-11-16

**Authors:** Laura Downham, Iman Jaafar, Mary Luz Rol, Victoria Nyawira Nyaga, Joan Valls, Armando Baena, Li Zhang, Marc J. Gunter, Marc Arbyn, Maribel Almonte

**Affiliations:** 1 Early Detection, Prevention and Infections Branch, International Agency for Research on Cancer, Lyon, France; 2 Department of Cancer Epidemiology, Scientific Institute of Public Health, Brussels, Belgium; 3 Cancer Epidemiology Research Program, Catalan Institute of Oncology (ICO), Idibell, Barcelona, Spain; 4 Department of Epidemiology and Biostatistics, School of Public Health, Imperial College London, London, UK; 5 Nutrition and Metabolism Branch, International Agency for Research on Cancer, Lyon, France; 6 Department of Human Structure and Repair, Faculty of Medicine and Health Sciences, Ghent University, Ghent, Belgium; 7 Department of Non-Communicable Diseases, World Health Organisation, Geneva, France

**Keywords:** Cancer epidemiology, Predictive markers

## Abstract

**Background:**

Cervical carcinogenesis is mediated by the HPV-E6 and E7 oncoproteins, considered as biomarkers usable in managing screen-positive women.

**Methods:**

We conducted a systematic review and meta-analysis assessing the accuracy of HPV-E6/E7-oncoprotein tests to detect underlying cervical-precancer and cancer. We included studies reporting data on oncoprotein test accuracy detecting cervical intraepithelial neoplasia grade 3 or worse. Random effects logistic regression models were applied for pooling absolute and relative accuracy.

**Results:**

Twenty-two studies were included. Sensitivity and specificity estimates ranged from 54.2% (95%CI: 45.2–63.0) to 69.5% (95%CI:60.8–76.9) and from 82.8% (95%CI: 50.4–95.8) to 99.1 (95%CI: 98.8–99.3), respectively in the population irrespective of HPV status. Higher sensitivity estimates ranging from 60.8% (95%CI: 49.6–70.9) to 75.5% (95%CI: 71.7–78.9) but lower specificity estimates ranging from 83.7% (95%CI: 76.1–89.3) to 92.1% (95%CI: 88.5–94.6) were observed in studies enrolling high-risk-HPV-positive women. Studies recruiting only HIV-positive women showed a pooled sensitivity of 46.9% (95%CI: 30.6–63.9) with a specificity of 98.0% (95%CI: 96.8–98.7).

**Conclusions:**

The high specificity of oncoprotein tests supports its use for triaging HPV-positive women. However, oncoprotein-negative women would not be recommended to undertake routine screening, requiring further follow-up. Large-scale and longitudinal studies are needed to further investigate the role of E6/E7-oncoprotein detection in predicting the risk of developing cervical pre-cancer and cancer.

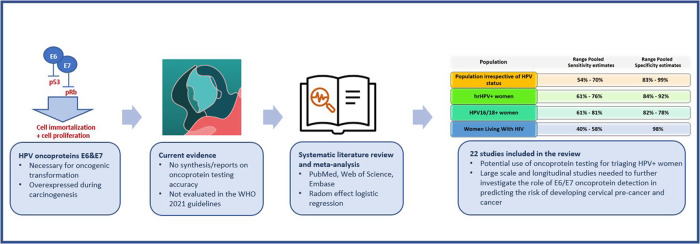

## Introduction

Cervical cancer is preventable, yet more than 600,000 new cases are diagnosed and approximately 350,000 deaths from the disease occur each year [1]. Cervical cancer is ranked as the fourth most common cancer worldwide and is, therefore, a major public health concern [2]. Persistent infection with carcinogenic Human Papilloma Virus (HPV) types is a necessary cause of cervical cancer [3]. HPV infection can lead to precancerous lesions such as cervical intraepithelial neoplasia 2, or 3 (CIN2, CIN3) which, if left untreated, can eventually progress to cervical cancer. Many women infected with HPV will naturally clear the infection within a year through their immune system, but in considerable proportion, the infection will persist for more than 2 years possibly leading to precancerous lesions and eventually transforming into cancer [4–7].

Most of the available HPV screening tests cannot differentiate between transient and persistent HPV infection and this is important for building an optimal and clinically relevant screening and follow-up approach. The 2021 guidelines from the World Health Organization (WHO) recommend using a high performance test for primary cervical cancer screening, such as HPV DNA assays [8]. However, due to the high prevalence of transient infections, HPV testing will increase the number of referrals to colposcopy and will generate unnecessary anxiety for women who are not at high risk of developing cervical cancer, highlighting the need for a second test to triage HPV positive women [9, 10]. Given the high global HPV prevalence, and especially in high-risk groups such as women living with HIV (WLWH) or in women from a low-and-middle-income country (LMIC), an accurate test is urgently needed to identify and triage those women who are at high risk of developing true disease. Current screening programmes in LMICs have limited resources and follow-up after the positivity of the first screening test can be challenging.

It is now well established that the viral oncoproteins E6 and E7 are necessary to promote and maintain cervical carcinogenesis and are both overexpressed during cervical transformation. Indeed, they have a synergic action and can activate many cancer hallmarks [11, 12]. The oncoprotein E6 has a major role in cell immortalization. It can bind to the tumour suppressor protein p53, which is usually activated in response to DNA damages in normal cells. This binding degrades the protein p53, which prevents the cell from undergoing apoptosis [13]. The oncoprotein E7 also interacts with numerous host proteins, and more specifically, it can bind to the cellular tumour suppressor, pRb [14, 15]. This binding to pRb leads to its degradation which prevents it from inactivating transcription factors and therefore favouring cell proliferation [16]. The overexpression of E6 and E7 leading to the accumulation of genetic errors in the cells over time in combination with viral gene deregulation expression, drives to the invasive cancer phenotype [12, 17, 18]. Oncoproteins E6 and E7 are, therefore, interesting candidates as markers of persistent HPV infection and associated pre-cancerous lesions. It is important to note that these oncoproteins E6 and E7 differ by HPV type and, therefore, tests must target oncoproteins from diverse oncogenic types to assure sufficient sensitivity. The clinical accuracy of E6 or E7 DNA and RNA assays have been evaluated in several comprehensive meta-analyses but the performance of tests targeting the E6 and E7 proteins has not been systematically reviewed yet [9, 19].

We aimed to synthesize the available and most up-to-date evidence on the accuracy of tests targeting HPV E6/E7 oncoproteins for CIN3+ (CIN2+) detection through a systematic review of the literature and meta-analysis.

## Methods

### Search strategy and selection criteria

The current systematic review includes a meta-analysis and was conducted following the Preferred Reporting Items for Systematic Reviews and Meta-analyses (PRISMA) requirements [20, 21]. The Population, Intervention, Comparator and Outcome (PICO) components were used to define the eligibility criteria (Supplementary Materials, page 3). Cohorts, case-control, cross-sectional diagnostic accuracy studies, and clinical trials were considered. Studies were included if they were reporting original data; if the disease of interest was cervical precancer (defined as cervical intraepithelial neoplasia of grade 2 or 3 or worse (CIN2 + /CIN3 + )); if they used a test targeting the E6 and/or the E7 oncoprotein; and if the gold standard for diagnosis was colposcopy guided histology or negative colposcopy. Articles were excluded if studies were in-vitro, animal-based or molecular; or if they assessed a proof of concept for the oncoprotein test development. Samples different from exfoliated cervical/vaginal cells such as sera or tissue samples were not considered.

A multi-database search was conducted, looking into PubMed, Embase and Web of Science for eligible publications in all languages without limitation of publication date until December 2021. The following search terms were used and combined: cervical cancer AND oncoproteins AND HPV AND screening. The Mesh function [MeSH] and Title and Abstract [tiab] were applied to refine the search (Supplementary Materials, pages 4–11). Reference lists of included articles and reviews on the topic were also manually screened. The grey literature was searched such as conference abstracts, thesis dissertations, reports, and local institution libraries to assess potential publication bias. More specifically, the online IARC library, EThOS (UK E-Theses Online Service), and the Open Grey website were thoroughly searched. Extensive searches were conducted to retrieve the full text of eligible articles, however, in case of unavailability, there were not included. In the case of multiple reports from the same study, the most recent one with the longest follow-up period or the one reporting the most comprehensive data was included. If unpublished data, researchers were systematically contacted to obtain as much information as possible. Summary estimates were sought for each included study. Titles and abstracts identified through the 3 electronic databases were screened by one reviewer (LD) and any questions or doubts were solved by discussion with a second reviewer (MLR).

### Data analysis

Titles and abstracts were screened against the eligibility criteria and for all eligible articles, full texts were obtained when possible. They were then independently examined in detail by two reviewers (LD and IJ) for their eligibility in the review. All papers excluded at this second stage of the selection process were documented along with the reasons for exclusion. Any questions and doubts were resolved through discussion or the intervention of a third reviewer (M. Arbyn). Moreover, studies requiring more calculation for data extraction were reviewed by a third reviewer (MLR). All identified references were stored into EndNote bibliographic software for further assessment and handling. Duplicate citations were removed manually. Hand-searching was preferred to the automatic de-duplicating option from EndNote as this could lead to removing articles that should not be deleted [22]. References were then transferred to Rayyan, an online application that allowed facilitating the screening process between reviewers [23]. For each included study, data was extracted by two independent reviewers (LD and IJ) and reported in a shared table (Supplementary materials, page 12. If any conflicts arose, they were resolved by discussion and submitted to M. Arbyn for final judgment, if necessary. Efforts were made to minimize reporting bias (Supplementary Materials, page 12).

The QUADAS-2 checklist was used to assess the quality of included diagnostic studies [24] (Supplementary Materials, pages 30–31).

Random effect logistic regression models were applied for pooling of sensitivity and specificity estimates and accounting for the intrinsic correlation between the log odds of the true and false positivity rate [25]. Results were displayed through forest plots. Separate meta-analyses were conducted for the outcomes CIN3+ (primary) and CIN2+. Analyses were stratified by the type of recruited study population, that is, screening, triage, colposcopy referral or convenience sample. If the results from the stratified analysis suggested some differences, a meta-regression was performed to formally explore differences with the covariate of interest. Heterogeneity statistic I^2^ was used to measure heterogeneity among studies. Studies targeting two different sources of populations—such as women coming from (i) a screening approach and (ii) women referred to colposcopy—were included in the convenience sample group. Of note, we invite the reader to interpret sub diamond results with caution when heterogeneity is high given the different design methodology of the included studies. Additional analyses were performed assessing some covariates as potential explanatory variables of accuracy: study population (population irrespective of HPV status, triage of hrHPV-positive or HPV16/18-positive), QUADAS quality items, countries’ incomes, and sample storage temperature. A separate analysis was conducted for studies including only women living with HIV (WLWH).

Relative estimates were also displayed through forest plots, comparing the performance of oncoprotein testing to that of comparator tests whenever reported, and we explicitly mentioned the comparator test in corresponding figures. These comparator tests included HPV DNA/RNA based tests; visual inspection tests or molecular-based tests.

If studies reported accuracy estimates separately for different populations such as (i) in the population irrespective of HPV status (ii) in hrHPV-positive women, or (iii) in HPV16/18-positive women; they were considered as three different studies in the analysis. Similarly, if studies reported accuracy estimates for different specimen types (clinician vs self-collected samples, stored in PreservCyt vs SurePath), data from clinician-collected samples in PreservCyt were extracted as the majority of included studies used this approach. When multiple HPV tests were used, we extracted the data for those HPV assays that are clinically validated [19]. Wherever HC2 (QIAGEN, Germantown, United States) and CareHPV (QIAGEN, Hilden, Germany) testing were used, HC2 testing was preferred as it has better accuracy than CareHPV for CIN3+ (CIN2+) detection [9]. Similarly, when cervical samples were collected with different devices, dry swab was the preferred collection approach rather than brush in liquid-based preservative medium. Finally, if a study reported accuracy estimates for two or more different oncoprotein tests, they were reported separately as two studies in the analysis.

All data were analysed using StataSE17 software.

### Role of the funding source

The funder of the study had no role in study design, data collection, data analysis, data interpretation, or writing of the report.

## Results

**Fig. 1:**
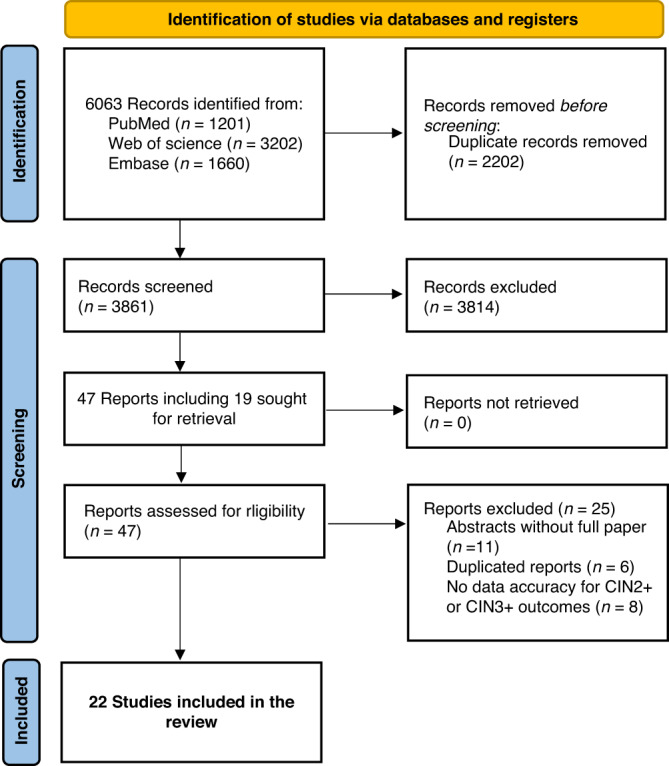
PRISMA* flow diagram. *PRISMA Preferred Reporting Items for Systematic review and Meta-analysis.

The search identified 6063 records which were eligible for title and abstract screening. Once duplicates were removed, 3861 records were screened from which 47 were screened in full text. Finally, 22 of those met the inclusion criteria and were therefore considered in the review for final analysis (Fig. 1) [26–47]. These studies were conducted between 1999 and 2020, and published between 2010 and 2020 (median 2017, IQR 2016–2019). Considering CIN2+ and CIN3+ endpoints together, six studies reported the accuracy of the oncoprotein test within a screening population, seven within a colposcopy referral population, and nine within a convenience sample (Supplementary Materials, pages 13–15). Two studies targeted only WLWH. In ten studies, the accuracy of the test was assessed in hrHPV+ women and six studies restricted the analysis to specifically HPV16/18+ women. Characteristics of the included studies are reported in Table 1 and all technical principles of oncoprotein tests are detailed in Supplementary Materials Pages 16–17.

**Table 1:** Study and population characteristics, and oncoprotein test accuracy for CIN2+ and/or CIN3+ detection.

	Study characteristics			Study population		HPV oncoprotein test			Sample collection		CIN2+		CIN3+	
Author, publication year	Study period	Study region(s)	Sample size	Study population*	Age range	Name, manufacturer	HPV type targets	Technique	Sampling	Medium	Se	Sp	Se	Sp
Population irrespective of HPV status (*n* = 11)														
Ara [26]	Jun 2018–Jun 2019	India	150	CR	21–65	OncoE6, Arbor vita	16/18	IC lateral flow	Clinician	Dry	52.2%	96.9%	81.8%	95.0%
Oliveira [21]	Jan–Sept 2017	Latin America	124	CS	25–64	OncoE6, Arbor vita	16/18	IC lateral flow	Clinician	LB	48.1%	100.0%	54.3%	100.0%
Oliveira [30]	Jan–Sept 2017	Latin America	124	CS	25–64	OncoE6, Arbor vita	16/18	IC lateral flow	SS	LB	31.6%	100.0%	35.7%	100.0%
Ferrera [31]	March–July 2014	Latin America	214	CS	30–64	OncoE6, Arbor vita	16/18	IC lateral flow	Clinician	Dry	NR	NR	56.4%	97.5%
Mariano [33]	NR	Latin America	447	CR	18–89	OncoE6, Arbor vita	16/18	IC lateral flow	Clinician	LB	49.2%	91.8%	59.2%	88.6%
Wu [43]	2013–2017	Asia	1717	CS	NR	OncoE6, Arbor vita	16/18	IC lateral flow	Clinician	Dry	68.9%	97.0%	74.5%	96.6%
Zhang, 2017	May 2014–Jan 2015	Asia	439	CR	25–65	OncoE6 Commercial kit, not specified	16/18	ELISA	Clinician	Dry	66.9%	97.1%	NR	NR
Yang [44]	NR	Asia, America	182	CS	NR	E6 test in-house	Non-type specific HPV E6 and E7 AB	Whole-cell ELISA	NR	LB	NR	NR	83.3%	69.3%
Kong [32]	Sept 2018–Sept 2019	Asia	205	CS	24–79	E7 protein assay, AMID Biotech	16/18	MPs-CEIA	Clinician	LB	70.6%	67.9%	67.4%	61.6%
Cuzick [29]	NR	UK	630	CR	NR	OncoHealth protein test E6/E7, OncoHealth	Non type specific HPV E6 AB	Whole-cell ELISA	Clinician	LB^a^	60.20%	44.9%	58.3%	43.8%
Cuzick [29]	NR	UK	630	CR	NR	OncoHealth protein test E6/E7, OncoHealth	Non type specific HPV E6 AB	Whole-cell ELISA	Clinician	LB^b^	52.3%	54.0%	55.2%	53.6%
Torres [40]	Aug 2014–Mar 2015	Latin America	389	S	18– ≥ 54	OncoE6, Arbor vita	16/18	IC lateral flow	SS	Dry	NR	NR	50.0%	99.2%
Valdez [42]	Oct 2010–Jun 2011	Asia	7421	S	25–65	OncoE6, Arbor vita	16/18/45	IC lateral flow	Clinician	Dry	42.0%	99.2%	54.4%	99.1%

hrHPV+ women (*n* = 12)														
Ferrera [31]	March–July 2014	Latin America	50	CS	30–64	OncoE6, Arbor vita	16/18	IC lateral flow	Clinician	Dry	NR	NR	96.8%	78.9%
Ferrera [31]	March–July 2014	Latin America	106	CS	30–64	OncoE6, Arbor vita	16/18	IC lateral flow	Clinician	Dry	NR	NR	55.6%	92.3%
Mariano [33]	NR	Latin America	131	CR	18–89	OncoE6, Arbor vita	16/18	IC lateral flow	Clinician	LB	76.0%	57.1%	80.8%	50.6%
Mariano [33]	NR	Latin America	265	CR	18–89	OncoE6, Arbor vita	16/18	IC lateral flow	Clinician	LB	49.2%	91.8%	NR	NR
Rezhake [36]	2014–2017	Asia	259	T	35–45	OncoE6E7, Arbor vita	16/18/31/35/45/33/52/58	IC lateral flow	Clinician	Dry	67.7%	89.5%	100.0%	85.9%
Rezhake [36]	2014–2017	Asia	259	T	35–45	OncoE6, Arbor vita	16/18	IC lateral flow	Clinician	Dry	51.6%	94.3%	80.0%	91.6%
Yu [45]	Apr 2014–Mar 2015	Asia	563	CS	21–75	OnoE6, Arbor vita	16/18	IC lateral flow	Clinician	Dry	86.4%	77.2%	87.8%	74.6%
Yu [45]	Apr 2014–Mar 2015	Asia	563	CS	21–75	OnoE6, Arbor vita	16/18	IC lateral flow	Clinician	Dry	77.8%	92.5%	77.7%	77.9%
Zhang, 2017	1999–2014	Asia	283	CR	35–45	OncoE6, Arbor vita	16/18	IC lateral flow	Clinician	LB	33.3%	92.0%	42.9%	90.9%
Zhang, 2017	1999–2014	Asia	245	CR	35–45	OncoE6, Arbor vita	16/18	IC lateral flow	Clinician	Dry	44.8%	94.0%	75.0%	91.6%
Schweizer [37]	NR	India Asia	91	CS	NR	OncoE6, Arbor vita	16/18/45	IC lateral flow	NR	Dry	NR	NR	68.0%	100.0%
Sellors, 2018	May 10–Jun 15 2007	Asia	38	CS	30–54	OncoE6, in-house	16/18	Western blot	Clinician	Dry	38.9%	95.0%	42.9%	83.9%
Agorastos [27]	Feb 2013– Jan 2015	Greece Germany	54	CR	30–60	In-house E7 test	16/18/45	Sandwich-ELISA-assay	Clinician	LB	100.0%	52.1%	NR	NR
Agorastos [27]	Feb 2013–Jan 2015	Greece Germany	144	CR	30–60	In-house E7 test	16/18/45	Sandwich-ELISA-assay	Clinician	LB	100.0%	49.2%	NR	NR
Shi [39]	Mar 2014–Feb 2015	Asia	450	CS	18–45	OncoE6E7, in-house	16/18	Western blot	Clinician	LB	71.3%	67.6%	NR	NR
Valdez [42]	Oct 2010–Jun 2011	Asia	1065	T	25–65	OncoE6, Arbor vita	16/18/45	IC lateral flow	Clinician	Dry	42.2%	95.1%	54.5%	94.6%
Torres-Ibarra [41]	Aug 2013–Feb 2016	Latin America	475	CR	30–64	OncoE6, Arbor vita	16/18	IC lateral flow	Clinician	LB	31.3%	83.6%	37.8%	83.5%
Qiao [35]	NR	Asia	1065	T	25–65	OncoE6, Arbor vita	16/18/45	IC lateral flow	Clinician	Dry	42.8%	94.3%	54.2%	93.8%
Qiao [35]	NR	Asia	1076	T	25–65	OncoE6, Arbor vita	16/18/45	IC lateral flow	Clinician	Dry	42.8%	94.2%	54.2%	93.8%
Qiao [35]	NR	Asia	1074	T	25–65	OncoE6, Arbor vita	16/18/45	IC lateral flow	SS	Dry	46.2%	96.0%	57.8%	95.5%
Qiao [35]	NR	Asia	1329	T	25–65	OncoE6, Arbor vita	16/18/45	IC lateral flow	SS	Dry	43.9%	96.1%	55.6%	95.5%

Women living with HIV														
Chibwesha [28]	Jan–Jun 2015	Africa	199	S	NR	OncoE6, Arbor vita	16/18	IC lateral flow	Clinician	LB	31.3%	99.4%	40.0%	98.3%
Ndizeye [34]	Jun–Dec 2017	Africa	679	S	25–65	OncoE6, Arbor vita	16/18	IC lateral flow	Clinician	Dry	42.1%	98.0%	58.3%	97.9%
Ndizeye [34]	Jun–Dec 2017	Africa	679	hrHPV+	25–65	OncoE6, Arbor vita	16/18	IC lateral flow	Clinician	Dry	42.1%	94.5%	58.3%	94.2%

*S* Screening, *T* Triage, *CR* Colposcopy Referral, *CS* Convenience Sample, *AB* Antibodies, *EIA* Enzyme Immunoassay, *HC2* Hybrid Capture, *IC* Immunochromatographic, *LB* Liquid-based, *LBC* Liquid-Based Cytology, *MPG* Multiplex Genotyping, *NR* Not Reported, *Se* Sensitivity, *Sp* Specificity, *SS* Self-Sample, *VIA* Visual Inspection with Acetic Acid.

^a^PreservCyt.

^b^SurePath.

**Fig. 2:**
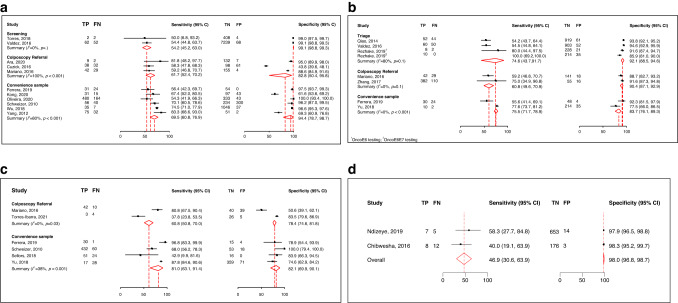
Accuracy of oncoprotein test for CIN3+ detection in the population irrespective of HPV status, hrHPV-positive women, in HPV16/18-positive women, and in women living with HIV. **a** Population irrespective of HPV status; **b** hrHPV-positive women; **c** HPV16/18-positive women; **d** Women Living with HIV; ^1^Testing with CncoE6; ^2^Testing with OncoE6E7; TP True Positives, FN False Negatives, TN True Negatives, FP False positives.

Among the 11 studies reporting the oncoprotein test accuracy for CIN3+ in the population irrespective of HPV status, the meta-analysis revealed sensitivity and specificity estimates of 54.2% (95%CI: 45.2–63.0) and 99·1% (95%CI: 98.8–99.3) for screening populations compared to 61.7% (95%CI: 52.4–70.2) and 82.8% (95%CI: 50.4–95.8) for colposcopy referral populations and finally 69.5% (95%CI: 60.8–76.9) and 94.4% (95%CI: 78.7–98.7) for convenience samples (Fig. 2a). These overall estimates did not significantly differ when stratified by population type as shown by the covariate analysis in the Supplementary Materials Page 18.

In hrHPV-positive women, the sensitivity was lower in colposcopy referral populations (60.8%) than in screening and convenience samples (74.6% and 75.5%, respectively), with specificities ranging from 83.7% to 92.1% across the different populations (Fig. 2b). However, these accuracy differences across the populations were not statistically significant (Supplementary Materials Page 19).

When restricted to HPV16/18+ women (Fig. 2c), the sensitivity was similar in the colposcopy referral group (60.8%, 95%CI: 50.8–70.0) and higher in the convenience sample (81%, 95%CI: 63.1–91.4) while the specificity was lower (78.4%, 95%CI: 74.6–81.8; 82.1%, 95%CI: 69.9–90.1, respectively) than in the population irrespective of HPV status and hrHPV-positive women. The covariate analysis did not show any significant accuracy differences between the two population groups (Supplementary Materials Page 20). Regarding the two studies including only WLWH, the pooled sensitivity estimate was 46.9% (95%CI: 30.6–63.9) with a corresponding specificity of 98.9% (95%CI: 96.8–98.7) (Fig. 2d).

In all populations, similar sensitivities were obtained with different sample storage temperatures, but the specificity increased from 68% with a storage at 4 °C to 98% with a storage at −60–80 °C as displayed in the Supplementary Materials Pages 21–23 (The meta-regression did not show significant differences in accuracy across the different groups of storage).

Most of the studies were conducted in upper-middle income countries (64%) with few in low-middle and upper-middle income countries (18%) as well as in (Upper-middle) high-income countries (18%). Stratification by income level did not explain study heterogeneity (Supplementary Materials, Page 24).

**Fig. 3:**
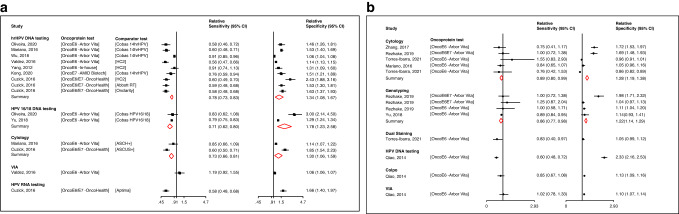
Accuracy of oncoprotein testing relative to other screening tests in the population irrespective of HPV status and in hrHPV-positive women. **a** Population irrespective of HPV status; **b** hrHPV-positive women. Results from a meta-regression using as a covariate the type of screening test. ASCH+ Atypical Squamous Cells where High-grade squamous intra-epithelial lesions cannot be excluded, ASCUS+ Atypical Squamous cells of Undetermined Significance, HC2 Hybrid Capture 2, HSIL High grade Squamous Intraepithelial Lesion, RT Real Time.

Eight studies were retrieved in which accuracy of the oncoprotein test to detect CIN3+ could be compared to that of other screening tests in the population irrespective of HPV status (Fig. 3a). When compared to hrHPV DNA, HPV16/18 DNA testing, mRNA testing, and cytology, oncoprotein testing was significantly less sensitive but more specific: relative sensitivity estimates of 0.78 (95%CI: 0.73–0.83); 0.71 (95%CI: 0.62–0.80); 0.58 (95%CI: 0.48–0.68); 0.73 (95%CI: 0.66–0.81), respectively, and relative specificity estimates of 1.34 (95%CI: 1.08–1.67); 1.78 (95%CI: 1.23–2.58); 1.66 (95%CI: 1.40–1.97); and 1.30 (95%CI: 1.06–1.59), respectively. Oncoprotein testing was 19% more sensitive and 6% more specific than visual inspection with acetic acid (VIA) for CIN3+ detection (relative sensitivity: 1.19, 95%CI: 0.92–1.55; relative specificity: 1.06, 95%CI: 1.06–1.07, *p* = 0.02).

The relative analysis from the six studies including hrHPV+ women showed similar results but were less marked (Fig. 3b). Oncoprotein testing was slightly less sensitive but more specific than cytology, genotyping (HPV DNA partial testing with LiPA 16/18 or Cobas HPV16/18; or extended genotyping testing with LiPA 8-types) and colposcopy with pooled relative sensitivity estimates of 0.89 (95%CI: 0.80–0.99), 0.86 (95%CI: 0.77–0.98), and 0.80 (95%CI: 0.59–1.07), respectively; and relative specificity of 1.28 (95%CI: 1.19–1.38), 1.22 (95%CI: 1.14–1.29), and 1.14 (95%CI: 1.04–1.26), respectively. In one study, oncoprotein testing was less sensitive than dual staining and HPV DNA testing (0.63, 95%CI: 0.40–0.97; 0.60, 95%CI: 0.48–0.72, respectively) but more specific (1.05, 95%CI: 0.99–1.12; 2.33, 95%CI: 2.16–2.53, respectively). Finally, oncoprotein testing showed a slightly better accuracy than VIA in one study with a relative sensitivity of 1.02 (95%CI: 0.78–1.33) and a relative specificity of 1.10 (95%CI: 1.07–1.14).

Results for the CIN2+ endpoint were similar to those of CIN3+ with the exception of oncoprotein testing that was found to be less sensitive than VIA in hrHPV-positive women (relative sensitivity: 0.52, 95%CI: 0.44–0.60, Supplementary Materials, Pages 25–29).

### Quality assessment using the QUADAS tool

Overall, there was a wide spectrum of patient selection approaches across the included studies (55%), reflecting the populations described from different sources, that is, women mainly enrolled from a clinical setting, gynaecological department, referred after a screen-positive results or needing colposcopy and therefore not derived from a primary screening population (Supplementary Materials, Pages 30–31).

We found an increased sensitivity with increased risk of bias in patient selection for CIN3+ detection (54%, 58%, and 68% in studies with low, moderate, and high risk of bias, respectively) (Supplementary Materials, Page 32).

Significantly increased risk of screening test bias with increased reported sensitivity for CIN3+ was found (58% in low, 69% in moderate, and 73% in high risk of bias, Supplementary Materials, Page 33).

Finally, there was an increased sensitivity with increased risk of bias in reference testing with overall 62% sensitivity in studies with a low-risk score and 72% sensitivity in studies with a moderate risk score (Supplementary Materials, Page 34).

## Discussion

The meta-analysis revealed a high specificity of oncoprotein testing, over 82% in the population irrespective of HPV status, in hrHPV-positive, in HPV16/18-positive and in WLWH. Moderate sensitivity estimates improved when the study population was restricted to hrHPV or HPV16/18-positive women. All estimates varied largely across studies with both sensitivity and specificity ranging from around 46.9% to 99.1%.

This wide observed variation in accuracy could be attributed to different factors such as type of oncoprotein test (targeting the E6 or E7 or both proteins, commercial or in-house developed test), sample storage and collection device. Accuracy estimates were improved when samples were stored between −60 °C and −80 °C. Sample collection device also varied between studies using dry Dacron swab or Cervex brush with a liquid-based medium but the lack of such data within the available studies did not allow further analyses considering these parameters. Studies from Kong et al., Schweizer et al., Wu et al., Yang et al., and Ara et al., reported sensitivities above 67% (range: 67.4% to 81.8%) [26, 32, 37, 43, 44]. A plausible reason for these high estimates could be explained by the study population. Indeed, most of these used a convenience sample of hrHPV-positive women including HPV16/18-positive women in the Kong study and enriched their population with cervical cancer cases in Schweizer, Wu, and Yang studies. Finally, women referred to colposcopy after an abnormal VIA result were included in one study [26]. Therefore, women included in these studies had higher probability of having high-grade lesions and thus were more prone to have elevated expression of oncoproteins with subsequent better detection from the oncoprotein testing. This is underpinned by the QUADAS analysis findings revealing high risk of bias in patient selection for Ara, Yang, Schweizer and Kong studies. It is worth noting that specificity in HPV16/18-positive women was lower than in the population irrespective of HPV status or in hrHPV-positive women (75% vs 94.2% vs 90.8%). This finding is consistent with the study from Rossi et al., showing that specificity decreases with high HPV prevalence. The results from the meta-regression that we conducted here showed that the slope is decreasing (−0.34, 95%CI: −1.69 -1.00) (Supplementary Materials Pages 35–36). Most of the included studies used the OncoE6 test, which is correlated to HPV types 16 and 18. OncoE6 testing could be used to triage HPV16/18-positive women perhaps to prioritise women positive for both HPD DNA and oncoprotein test compared to oncoprotein-negative women.

Our results showed that oncoprotein testing was significantly more specific than HPV DNA and mRNA testing (relative specificity of 1.34 and 1.66, respectively). This finding is consistent with results from several studies reporting stronger correlations between E6 oncoprotein expression and severity of the cervical lesion than the correlation between HPV positivity and lesion severity [37, 38, 48]. Wu et al. reported a positive association between HPV DNA viral load and expression of E6 oncoprotein in (pre-)cancer lesions as well as consistency regarding oncoprotein E6 positivity and hrHPV viral load and increased disease severity [43]. These findings highlight the potential of using oncoprotein detection as a marker of disease severity and progression towards CIN3 or cancer.

It has been shown that there is an increased number of cells with integrated HPV genome in cells progressing to malignancy [49]. A study from the Cancer Genome Atlas showed that HPV was integrated in all HPV18-positive cases and in 76% of HPV16-positive cases [50]. From our meta-analysis results, oncoprotein testing appears to have a low sensitivity compared to HPV DNA testing. The main hypothesis relies on the viral HPV DNA, which would have not been integrated within the host genome in precancerous lesions not progressing to cervical cancer. Therefore, the oncoproteins E6/E7 are not expressed -or are undetectable- and the oncoprotein test cannot detect them as the oncoproteins are only highly expressed in true pre-cancerous cells. Although it has been shown that integration is associated with progression, the integration process does not mean that episomal HPV disappears and, therefore, totally explains drop in the sensitivity of HPV testing. This is only a hypothesis and will require further studies to investigate. In some studies, false positivity of the oncoprotein test was high, possibly explained by technical limitations of the tests themselves as well as sampling methods, the number and quality of collected cells and the storage process [29, 32, 44]. However, we are aware that the comparison in sensitivity between oncoproteins and HPV DNA testing has not a strong rationale given that HPV infection is a necessary condition for the disease. The additional interest here includes triaging WLWH or immunocompromised HPV-positive women in general and especially in screen and treat schemes to avoid unnecessary treatment. Indeed, WLWH come back to care more regularly than HIV-negative women, thus the opportunity to be reassessed more often.

To date, the gold standard test for cervical cancer diagnosis is histological examination which provides clinical information such as stage of the disease. However, whether the cervical lesion will be more likely to progress, or regress, is still complex to predict. Hence, the detection of E6/E7 oncoproteins could add another level of information regarding the potential progress to carcinogenic phenotype and help identify women at higher risk of developing true disease.

Most of the studies were conducted in upper-middle income countries with a few in low-middle income countries or high-income countries. The test accuracy did not appear to be compromised by the income country level, revealing the possibility of performing the test in such resource limited settings.

This meta-analysis is the first reporting on the accuracy of oncoprotein testing. Data were extracted and revised by different reviewers and a rigorous QUADAS analysis was conducted. The literature search was neither restricted to language nor to publication date.

It is important to note that given the recruitment strategy, all included studies reporting accuracy in hrHPV-positive women could not be assessed as a triage setting. Indeed, not all included women were hrHPV-positive, and all did not have one or more triage test(s) as different diagnostic algorithms were applied. Similarly, studies including a convenience sample could not be assessed as a screening setting given that most of included women were an arbitrary case-mix derived from primary screening and from a clinical setting and referred from screen-positive results. Our review was restricted to cross-sectional outcomes that is, sensitivity and specificity of the oncoprotein test against the reference test with no available longitudinal data to evaluate. Finally, there was too much variation in reporting age, which precluded us to assess it as covariate of oncoprotein accuracy.

More research is needed to assess whether oncoprotein testing can distinguish between HPV driven lesions with higher potential of carcinogenesis and lesions with poor progression potential. The study from Zhang et al. reported on the role of the OncoE6 testing as a triage method for HPV positive women, to predict the risk of developing CIN3+. It showed that women with persistent hrHPV infection had 40 times the odds (95%CI = 11.8–135.5) of expressing the E6 oncoprotein compared to women with incident HPV infection during the 15-year follow-up and that there was an increased risk of subsequent HPV DNA persistence in women who tested positive for OncoE6 compared to women who tested negative (adjusted OR = 21.2, 95%CI = 5.2–86.4). Finally, more research focusing on the regressions rates of oncoprotein negative CIN3 lesions would help understanding of the longitudinal safety of oncoprotein testing.

In conclusion, this meta-analysis reveals a high specificity of oncoprotein testing across all groups (population irrespective of HPV status, hrHPV-positive, HPV16/18-positive, and WLWH), with moderate sensitivity estimates. The corresponding sensitivity estimates were comparable or higher to those of cytology and VIA for CIN3+ detection in primary screening. Oncoprotein testing may be useful for triaging HPV-positive women. However, the role of oncoprotein test should be further explored in populations such as WLWH for whom high specific screening and triage tests may be needed.

### Supplementary information

Supplementary materials to the manuscript entitled "Accuracy of HPV E6/E7 oncoprotein tests to detect high-grade cervical lesions: a systematic literature review and meta-analysis

## Data Availability

The datasets generated during and/or analysed during the current study are available from the corresponding author on reasonable request.
