# Supplementary material for: Accuracy of HPV E6/E7 oncoprotein tests to detect high-grade cervical lesions: a systematic literature review and meta-analysis
Source: Br J Cancer. 2023 Nov 16;130(4):517–25. doi: 10.1038/s41416-023-02490-w (PMC10876647; doi:10.1038/s41416-023-02490-w)
Supplement: Supplementary file 1 — Supplementary materials to the manuscript entitled "Accuracy of HPV E6/E7 oncoprotein tests to detect high-grade cervical lesions: a systematic literature review and meta-analysis [file 41416_2023_2490_MOESM1_ESM.docx]

**SUPPLEMENTARY MATERIALS TO THE MANUSCRIPT**

**Accuracy of HPV E6/E7 oncoprotein tests to detect high-grade cervical lesions: a systematic literature review and meta-analysis**

Laura Downham^1^, Iman Jaafar^2^, Mary Luz Rol^1^, Victoria Nyawira Nyaga^2^, Joan Valls^1,3^, Armando Baena^1^, Li Zhang^1^, Marc Gunter^4,5^, Marc Arbyn^2,6^, Maribel Almonte^1,7^

^1^Department of Early Detection, Prevention and Infections Branch, International Agency for Research on Cancer, Lyon 69007, France (L Downham MSc; ML Rol PhD; J Valls PhD; A Baena PhD; L Zhang PhD; M Almonte PhD)

^2^Department of Cancer Epidemiology, Scientific Institute of Public Health, Brussels, Belgium (M Arbyn DrTM&H; I Jaafar MSc, V Nyaga PhD)

^3^Cancer Epidemiology Research Program, Catalan Institute of Oncology (ICO), Idibell, Barcelona, Spain (J Valls PhD)

^4^Department of Epidemiology and Biostatistics, School of Public Health, Imperial College London, United Kingdom (M Gunter, PhD)

^5^Department of Nutrition and Metabolism, International Agency for Research on Cancer, Lyon, France

^6^ Department of Human Structure and Repair, Faculty of Medicine and Health Sciences, Ghent University, Belgium (M Arbyn DrTM&H)

^7^Department of Non-Communicable Diseases, World Health Organisation, Geneva, France

**Table of contents**

[**1** **PICO components** 3](#_Toc144382720)

[**2** **Literature search strategy** 4](#_Toc144382721)

[**3** **Study data extraction** 12](#_Toc144382722)

[**4** **Bias consideration** 12](#_Toc144382723)

[**5** **Description of included study populations and available outcomes for accuracy** 13](#_Toc144382724)

[**6** **Description of all oncoprotein tests used in the 22 included studies** 16](#_Toc144382725)

[**7** **Meta-regression analyses exploring the population (screening vs colposcopy referral vs convenience sample) as covariate of interest** 18](#_Toc144382726)

[**8** **Accuracy of oncoprotein testing according to sample storage temperatures** 21](#_Toc144382727)

[**9** **Accuracy of oncoprotein testing in studies with different country income levels** 24](#_Toc144382728)

[**10** **Accuracy of oncoprotein testing to detect CIN2+** 25](#_Toc144382729)

[**11** **Quality assessment of the included studies reporting on the accuracy of oncoprotein testing** 30](#_Toc144382730)

[**12** **Specificity of oncoprotein testing as function of HPV prevalence** 35](#_Toc144382731)

# **PICO components**

- **P_1_**opulation: Women attending cervical cancer screening; screened-positive women to be triaged; or women referred to colposcopy
- **P_2_**opulation: Women living with HIV (WLHIV)
- **I**ntervention: HPV test targeting E6 and/or E7 oncoprotein(s) for cervical cancer screening and/or triage
- **C**omparator: Other tests: cytology, high-risk (hr) HPV tests, VIA, HPV genotyping, immunocyto-chemistry identifying the proteins p16 and K-i67 (dual staining)
- **O_1_**utcome: Absolute sensitivity and specificity for CIN3+ (CIN2+) detection of the index test
- **O_2_**utcome: Relative sensitivity and specificity for CIN3+ (CIN2+) detection of the index test versus the comparator tests

# **Literature search strategy**

**Supplementary table 1. Research question in PubMed database**

| **Search number** | **Query** | **Search Details** |
| --- | --- | --- |
| 52 | #14 AND #32 AND #39 AND #51 | ("uterine cervical neoplasms"[MeSH Terms] OR "uterine cervical dysplasia"[MeSH Terms] OR "cervical intraepithelial neoplasia"[MeSH Terms] OR "uterine cervical dysplasia"[MeSH Terms] OR "cervical dysplas*"[Title/Abstract] OR "cervical intraepithelial neoplas*"[Title/Abstract] OR "cervical neoplas*"[Title/Abstract] OR "cervical cancer*"[Title/Abstract] OR "cervix neoplas*"[Title/Abstract] OR "cervix cancer*"[Title/Abstract] OR "cervix dysplas*"[Title/Abstract] OR "uterine cervical neoplas*"[Title/Abstract] OR (("CIN"[Title/Abstract] OR "cin i"[Title/Abstract] OR "cin ii"[Title/Abstract] OR "cin iii"[Title/Abstract] OR "cin 1"[Title/Abstract] OR "cin 2"[Title/Abstract] OR "cin 3"[Title/Abstract] OR "CINI"[Title/Abstract] OR "CINII"[Title/Abstract] OR "CINIII"[Title/Abstract] OR "CIN1"[Title/Abstract] OR "CIN2"[Title/Abstract] OR "CIN3"[Title/Abstract]) AND ("cervix*"[Title/Abstract] OR "cervical*"[Title/Abstract] OR "uterine*"[Title/Abstract]))) AND ("papillomavirus e7 proteins"[MeSH Terms] OR "papillomavirus e7 protein*"[Title/Abstract] OR "E6E7"[Title/Abstract] OR "e6 e7"[Title/Abstract] OR "E6"[Title/Abstract] OR "E7"[Title/Abstract] OR "OncoE6"[Title/Abstract] OR "onco e6"[Title/Abstract] OR ("Onco"[All Fields] AND "E7"[Title/Abstract]) OR ("OncoE6"[Title/Abstract] AND "E7"[Title/Abstract]) OR "oncogene protein*"[Title/Abstract] OR "oncoprotein*"[Title/Abstract] OR "viral protein*"[Title/Abstract] OR "hpv protein*"[Title/Abstract] OR "hpv oncoprotein*"[Title/Abstract] OR "protein assay*"[Title/Abstract] OR "protein test*"[Title/Abstract]) AND ("Papillomaviridae"[MeSH Terms] OR "papillomavirus infections"[MeSH Terms] OR "Papillomaviridae"[Title/Abstract] OR "papillomavirus infection*"[Title/Abstract] OR "human papillomavirus"[Title/Abstract] OR "HPV"[Title/Abstract]) AND ("early detection of cancer"[MeSH Terms] OR "triage"[MeSH Terms] OR "mass screening"[MeSH Terms] OR "diagnos*"[Title/Abstract] OR "screening*"[Title/Abstract] OR "screen"[Title/Abstract] OR "triage"[Title/Abstract] OR "risk stratification"[Title/Abstract] OR "management"[Title/Abstract] OR "detection*"[Title/Abstract] OR "detect"[Title/Abstract]) |
| 51 | #40 OR #41 OR #42 OR #43 OR #44 OR #45 OR #46 OR #47 OR #48 OR #49 OR #50 | "early detection of cancer"[MeSH Terms] OR "triage"[MeSH Terms] OR "mass screening"[MeSH Terms] OR "diagnos*"[Title/Abstract] OR "screening*"[Title/Abstract] OR "screen"[Title/Abstract] OR "triage"[Title/Abstract] OR "risk stratification"[Title/Abstract] OR "management"[Title/Abstract] OR "detection*"[Title/Abstract] OR "detect"[Title/Abstract] |
| 50 | detect[Title/Abstract] | "detect"[Title/Abstract] |
| 49 | detection*[Title/Abstract] | "detection*"[Title/Abstract] |
| 48 | management[Title/Abstract] | "management"[Title/Abstract] |
| 47 | risk stratification[Title/Abstract] | "risk stratification"[Title/Abstract] |
| 46 | triage[Title/Abstract] | "triage"[Title/Abstract] |
| 45 | screen[Title/Abstract] | "screen"[Title/Abstract] |
| 44 | screening*[Title/Abstract] | "screening*"[Title/Abstract] |
| 43 | diagnos*[Title/Abstract] | "diagnos*"[Title/Abstract] |
| 42 | Mass screening[MeSH Terms] | "mass screening"[MeSH Terms] |
| 41 | triage[MeSH Terms] | "triage"[MeSH Terms] |
| 40 | early detection of cancer[MeSH Terms] | "early detection of cancer"[MeSH Terms] |
| 39 | #33 OR #34 OR #35 OR #36 OR #37 OR #38 | "Papillomaviridae"[MeSH Terms] OR "papillomavirus infections"[MeSH Terms] OR "Papillomaviridae"[Title/Abstract] OR "papillomavirus infection*"[Title/Abstract] OR "human papillomavirus"[Title/Abstract] OR "HPV"[Title/Abstract] |
| 38 | HPV[Title/Abstract] | "HPV"[Title/Abstract] |
| 37 | human papillomavirus[Title/Abstract] | "human papillomavirus"[Title/Abstract] |
| 36 | Papillomavirus infection*[Title/Abstract] | "papillomavirus infection*"[Title/Abstract] |
| 35 | Papillomaviridae[Title/Abstract] | "Papillomaviridae"[Title/Abstract] |
| 34 | Papillomavirus infections[MeSH Terms] | "papillomavirus infections"[MeSH Terms] |
| 33 | Papillomaviridae[MeSH Terms] | "papillomaviridae"[MeSH Terms] |
| 32 | #15 OR #16 OR #17 OR #18 OR #19 OR #20 OR #21 OR #22 OR #23 OR #24 OR #25 OR #26 OR #27 OR #28 OR #29 OR #30 OR #31 | "papillomavirus e7 proteins"[MeSH Terms] OR "papillomavirus e7 protein*"[Title/Abstract] OR "E6E7"[Title/Abstract] OR "e6 e7"[Title/Abstract] OR "E6"[Title/Abstract] OR "E7"[Title/Abstract] OR "OncoE6"[Title/Abstract] OR "onco e6"[Title/Abstract] OR ("Onco"[All Fields] AND "E7"[Title/Abstract]) OR ("OncoE6"[Title/Abstract] AND "E7"[Title/Abstract]) OR "oncogene protein*"[Title/Abstract] OR "oncoprotein*"[Title/Abstract] OR "viral protein*"[Title/Abstract] OR "hpv protein*"[Title/Abstract] OR "hpv oncoprotein*"[Title/Abstract] OR "protein assay*"[Title/Abstract] OR "protein test*"[Title/Abstract] |
| 31 | protein test*[Title/Abstract] | "protein test*"[Title/Abstract] |
| 30 | protein assay*[Title/Abstract] | "protein assay*"[Title/Abstract] |
| 29 | HPV oncoprotein*[Title/Abstract] | "hpv oncoprotein*"[Title/Abstract] |
| 28 | HPV protein*[Title/Abstract] | "hpv protein*"[Title/Abstract] |
| 27 | viral protein*[Title/Abstract] | "viral protein*"[Title/Abstract] |
| 26 | oncoprotein*[Title/Abstract] | "oncoprotein*"[Title/Abstract] |
| 25 | oncogene protein*[Title/Abstract] | "oncogene protein*"[Title/Abstract] |
| 24 | OncoE6[tiab] AND E7[tiab] | "OncoE6"[Title/Abstract] AND "E7"[Title/Abstract] |
| 23 | Onco E7[Title/Abstract] | "Onco"[All Fields] AND "E7"[Title/Abstract] |
| 22 | Onco E6[Title/Abstract] | "onco e6"[Title/Abstract] |
| 21 | OncoE6[Title/Abstract] | "OncoE6"[Title/Abstract] |
| 20 | E7[Title/Abstract] | "E7"[Title/Abstract] |
| 19 | E6[Title/Abstract] | "E6"[Title/Abstract] |
| 18 | E6/E7[Title/Abstract] | "e6 e7"[Title/Abstract] |
| 17 | E6E7[Title/Abstract] | "E6E7"[Title/Abstract] |
| 16 | papillomavirus E7 protein*[Title/Abstract] | "papillomavirus e7 protein*"[Title/Abstract] |
| 15 | Papillomavirus E7 proteins[MeSH Terms] | "papillomavirus e7 proteins"[MeSH Terms] |
| 14 | #1 OR #2 OR #3 OR #4 OR #5 OR #6 OR #7 OR #8 OR #9 OR #10 OR #11 OR #12 OR #13 | "uterine cervical neoplasms"[MeSH Terms] OR "uterine cervical dysplasia"[MeSH Terms] OR "cervical intraepithelial neoplasia"[MeSH Terms] OR "uterine cervical dysplasia"[MeSH Terms] OR "cervical dysplas*"[Title/Abstract] OR "cervical intraepithelial neoplas*"[Title/Abstract] OR "cervical neoplas*"[Title/Abstract] OR "cervical cancer*"[Title/Abstract] OR "cervix neoplas*"[Title/Abstract] OR "cervix cancer*"[Title/Abstract] OR "cervix dysplas*"[Title/Abstract] OR "uterine cervical neoplas*"[Title/Abstract] OR (("CIN"[Title/Abstract] OR "cin i"[Title/Abstract] OR "cin ii"[Title/Abstract] OR "cin iii"[Title/Abstract] OR "cin 1"[Title/Abstract] OR "cin 2"[Title/Abstract] OR "cin 3"[Title/Abstract] OR "CINI"[Title/Abstract] OR "CINII"[Title/Abstract] OR "CINIII"[Title/Abstract] OR "CIN1"[Title/Abstract] OR "CIN2"[Title/Abstract] OR "CIN3"[Title/Abstract]) AND ("cervix*"[Title/Abstract] OR "cervical*"[Title/Abstract] OR "uterine*"[Title/Abstract])) |
| 13 | ((CIN[tiab] OR CIN I[tiab] OR CIN II[tiab] OR CIN III[tiab] OR CIN 1[tiab] OR CIN 2[tiab] or CIN 3[tiab] OR CINI[tiab] OR CINII[tiab] OR CINIII[tiab] OR CIN1[tiab] OR CIN2[tiab] OR CIN3[tiab]) AND (cervix*[tiab] OR cervical*[tiab] OR uterine*[tiab])) | ("CIN"[Title/Abstract] OR "cin i"[Title/Abstract] OR "cin ii"[Title/Abstract] OR "cin iii"[Title/Abstract] OR "cin 1"[Title/Abstract] OR "cin 2"[Title/Abstract] OR "cin 3"[Title/Abstract] OR "CINI"[Title/Abstract] OR "CINII"[Title/Abstract] OR "CINIII"[Title/Abstract] OR "CIN1"[Title/Abstract] OR "CIN2"[Title/Abstract] OR "CIN3"[Title/Abstract]) AND ("cervix*"[Title/Abstract] OR "cervical*"[Title/Abstract] OR "uterine*"[Title/Abstract]) |
| 12 | uterine cervical neoplas*[Title/Abstract] | "uterine cervical neoplas*"[Title/Abstract] |
| 11 | cervix dysplas*[Title/Abstract] | "cervix dysplas*"[Title/Abstract] |
| 10 | cervix cancer*[Title/Abstract] | "cervix cancer*"[Title/Abstract] |
| 9 | cervix neoplas*[Title/Abstract] | "cervix neoplas*"[Title/Abstract] |
| 8 | cervical cancer*[Title/Abstract] | "cervical cancer*"[Title/Abstract] |
| 7 | cervical neoplas*[Title/Abstract] | "cervical neoplas*"[Title/Abstract] |
| 6 | cervical intraepithelial neoplas*[Title/Abstract] | "cervical intraepithelial neoplas*"[Title/Abstract] |
| 5 | cervical dysplas*[Title/Abstract] | "cervical dysplas*"[Title/Abstract] |
| 4 | cervix dysplasia[MeSH Terms] | "uterine cervical dysplasia"[MeSH Terms] |
| 3 | cervical intraepithelial neoplasia[MeSH Terms] | "cervical intraepithelial neoplasia"[MeSH Terms] |
| 2 | uterine cervical dysplasia[MeSH Terms] | "uterine cervical dysplasia"[MeSH Terms] |
| 1 | uterine cervical neoplasms[MeSH Terms] | "uterine cervical neoplasms"[MeSH Terms] |

**Supplementary table 2. Research question in Embase database**

| **Search number** | **Query** |
| --- | --- |
| #44 | #11 AND #27 AND #33 AND #43 |
| #43 | #34 OR #35 OR #36 OR #37 OR #38 OR #39 OR #40 OR #41 OR #42 |
| #42 | 'detect':ab,ti |
| #41 | 'detection*':ab,ti |
| #40 | 'management':ab,ti |
| #39 | 'risk stratification':ab,ti |
| #38 | 'screen':ab,ti |
| #37 | 'screening*':ab,ti |
| #36 | 'diagnos*':ab,ti |
| #35 | 'triage':ab,ti |
| #34 | 'early cancer diagnosis'/exp OR 'mass screening'/exp |
| #33 | #28 OR #29 OR #30 OR #31 OR #32 |
| #32 | 'hpv':ab,ti |
| #31 | 'human papillomavirus':ab,ti |
| #30 | 'papillomavirus infection*':ab,ti |
| #29 | 'papillomaviridae':ab,ti |
| #28 | 'papillomaviridae'/exp OR 'papillomavirus infection'/exp |
| #27 | #12 OR #13 OR #14 OR #15 OR #16 OR #17 OR #18 OR #19 OR #20 OR #21 OR #22 OR #23 OR #24 OR #25 OR #26 |
| #26 | 'protein test*':ab,ti |
| #25 | 'protein assay*':ab,ti |
| #24 | 'hpv oncoprotein*':ab,ti |
| #23 | 'hpv protein*':ab,ti |
| #22 | 'viral protein*':ab,ti |
| #21 | 'oncoprotein*':ab,ti |
| #20 | 'oncogene protein*':ab,ti |
| #19 | 'onco e6':ab,ti |
| #18 | 'oncoe6':ab,ti |
| #17 | 'e7':ab,ti |
| #16 | 'e6':ab,ti |
| #15 | 'e6 e7':ab,ti |
| #14 | 'e6e7':ab,ti |
| #13 | 'papillomavirus e7 protein*':ab,ti |
| #12 | 'protein e7'/exp OR 'protein e6'/exp |
| #11 | #1 OR #2 OR #3 OR #4 OR #5 OR #6 OR #7 OR #8 OR #9 OR #10 |
| #10 | ('cin':ab,ti OR 'cin i':ab,ti OR 'cin ii':ab,ti OR 'cin iii':ab,ti OR 'cin 1':ab,ti OR 'cin 2':ab,ti OR 'cin 3':ab,ti OR 'cini':ab,ti OR 'cinii':ab,ti OR 'ciniii':ab,ti OR 'cin1':ab,ti OR 'cin2':ab,ti OR 'cin3':ab,ti) AND ('cervix*':ab,ti OR 'cervical*':ab,ti OR 'uterine*':ab,ti) |
| #9 | 'uterine cervical neoplas*':ab,ti |
| #8 | 'cervix dysplas*':ab,ti |
| #7 | 'cervix cancer*':ab,ti |
| #6 | 'cervix neoplas*':ab,ti |
| #5 | 'cervical cancer*':ab,ti |
| #4 | 'cervical neoplas*':ab,ti |
| #3 | 'cervical intraepithelial neoplas*':ab,ti |
| #2 | 'cervical dysplas*':ab,ti |
| #1 | 'uterine cervix cancer'/exp OR 'uterine cervix tumor'/exp OR 'uterine cervix carcinoma in situ'/exp OR 'uterine cervix dysplasia'/exp |

**Supplementary table 3. Research question in Web of Science database**

| **Search number** | **Query** |
| --- | --- |
| #49 | (((#16) AND #33) AND #38) AND #48 |
| #48 | ((((((((#39) OR #40) OR #41) OR #42) OR #43) OR #44) OR #46) OR #45) OR #47 |
| #47 | TS=(detect) |
| #46 | TS=(detection*) |
| #45 | TS=(management) |
| #44 | TS=(risk stratification) |
| #43 | TS=(screen) |
| #42 | TS=(screening*) |
| #41 | TS=(diagnos*) |
| #40 | TS=(triage) |
| #39 | TS=(early detection of cancer) |
| #38 | (((#34) OR #35) OR #36) OR #37 |
| #37 | TS=(HPV) |
| #36 | TS=(human papillomavirus) |
| #35 | TS=(papillomavirus infection*) |
| #34 | TS=(papillomaviridae) |
| #33 | (((((((((((((((#17) OR #18) OR #19) OR #20) OR #21) OR #22) OR #23) OR #24) OR #25) OR #26) OR #27) OR #28) OR #29) OR #30) OR #31) OR #32 |
| #32 | TS=(protein test*) |
| #31 | TS=(protein assay*) |
| #30 | TS=(HPV oncoprotein*) |
| #29 | TS=(HPV protein*) |
| #28 | TS=(viral protein*) |
| #27 | TS=(oncoprotein*) |
| #26 | TS=(oncogene protein*) |
| #25 | TS=(OncoE6 E7) |
| #24 | TS=(Onco E7) |
| #23 | TS=(Onco E6) |
| #22 | TS=(OncoE6) |
| #21 | TS=(E7) |
| #20 | TS=(E6) |
| #19 | TS=(E6 E7) |
| #18 | TS=(E6E7) |
| #17 | TS=(papillomavirus E7 protein*) |
| #16 | ((((((((((((#1) OR #2) OR #3) OR #4) OR #5) OR #6) OR #7) OR #8) OR #9) OR #10) OR #11) OR #12) OR #15 |
| #15 | (#13) AND #14 |
| #14 | ((TS=(cervix*)) OR TS=(cervical*)) OR TS=(uterine*) |
| #13 | ((((((((((((TS=(CIN)) OR TS=(CIN I)) OR TS=(CIN II)) OR TS=(CIN III)) OR TS=(CIN 1)) OR TS=(CIN 2)) OR TS=(CIN 3)) OR TS=(CINI)) OR TS=(CINII)) OR TS=(CINIII)) OR TS=(CIN1)) OR TS=(CIN2)) OR TS=(CIN3) |
| #12 | TS=(uterine cervical neoplas*) |
| #11 | TS=(cervix dysplas*) |
| #10 | TS=(cervix cancer*) |
| #9 | TS=(cervix neoplas*) |
| #8 | TS=(cervical cancer*) |
| #7 | TS=(cervical neoplas*) |
| #6 | TS=(cervical intraepithelial neoplas*) |
| #5 | TS=(cervical dysplas*) |
| #4 | TS=(cervix dysplasia) |
| #3 | TS=(cervical intraepithelial neoplasia) |
| #2 | TS=(uterine cervical dysplasia) |
| #1 | TS=(uterine cervical neoplasms) |

# **Study data extraction**

- General article information: author, title, publication year and journal
- Study information: study design, study region(s) and period, overall aim, inclusion/exclusion criteria, sample size
- Study population characteristics: study population (screening/mixed), age range, mean or median age when available, HIV status when reported
- Information regarding the HPV oncoprotein test (index test): oncoprotein positivity, name of the test, HPV types targeted by the test, test manufacturer, test technique (ELISA, Western blot, or Immunochromatographic lateral flow)
- Name of the comparative tests
- Information regarding the cervical sample: storage, sample collection approach (collected by a clinician or self-collected), sampling method (dry or liquid-based), sample collection device (cervical swab, Cervex brush etc.)
- Information reported to minimise diagnosis related bias: pathologist for histology examination (e.g., blinded to initial testing), definition of free from disease
- Information regarding the outcome: outcome definition (CIN2+/CIN3+ on histology), sensitivity, specificity, setting for accuracy of the index test and comparative test(s)
- The number of true positives, false positives, true negatives, and false negative were extracted or calculated for each included study, for both the index test and comparative test(s).

# **Bias consideration**

Efforts were made to minimize reporting bias such as publication bias, selective outcome reporting, ascertainment bias, as well as inclusion bias. Indeed, a protocol was written and reviewed before starting the review; the search was documented; a review team was established; there was neither restriction regarding the publication date, nor regarding the language; the grey literature was searched; emails were sent to authors whenever information was needed, and transparent reporting was done following the PRISMA reporting guidelines.

.

# **Description of included study populations and available outcomes for accuracy**

**Supplementary table 4. Summary of the 22 study populations and outcomes reported**

| **Study, publication year** | **Recruited population** | **Population for analysis** | **Oncoprotein accuracy in all women** | | **Oncoprotein accuracy in hrHPV+** | | **Oncoprotein accuracy in HPV16/18+** | |
| --- | --- | --- | --- | --- | --- | --- | --- | --- |
|  |  |  | **CIN2+** | **CIN3+** | **CIN2+** | **CIN3+** | **CIN2+** | **CIN3+** |
| Qiao, 2013 | Screening (screening with OncoE6 and HPV DNA QIAGEN testing) | hrHPV+ |  |  | ✓ | ✓ |  |  |
| Torres, 2018 | Screening (screening with OncoE6, HPV DNA QIAGEN testing and HPV genotyping Papillocheck testing) | Population irrespective of HPV status |  | ✓ |  |  |  |  |
| Valdez, 2016 | Screening (screening with OncoE6, HPV DNA QIAGEN testing and VIA) | 1. Population irrespective of HPV status 2. hrHPV+ | ✓ | ✓ | ✓ | ✓ |  |  |
| Ara, 2020 | 1. Colposcopy referral: Abnormal or normal VIA 2. Colposcopy referral: Abnormal or normal Pap test | Population irrespective of HPV status | ✓ | ✓ |  |  |  |  |
| Cuzick, 2016 | Colposcopy Referral: Abnormal screening result | Population irrespective of HPV status | ✓ |  |  |  |  |  |
| Oliveira, 2020 | 1. Normal cytology 2. Convenience sample of CIN2, CIN3 and CC | Population irrespective of HPV status | ✓ |  |  |  |  |  |
| Ferrera, 2019 | 1. Convenience sample of hrHPV+ and hrHPV- women 2. Colposcopy referral: Abnormal cytology (HSIL or AGC) and histology-confirmed CIN2+- Ask Armando data for this group only | 1. Population irrespective of HPV status 2. hrHPV+ 3. HPV16/18+ |  | ✓ |  | ✓ |  | ✓ |
| Mariano, 2016 | Colposcopy Referral: ASCH+ on Pap test | 1. Population irrespective of HPV status 2. hrHPV+ 3. HPV16/18+ | ✓ |  | ✓ |  | ✓ | ✓ |
| Wu, 2018 | 1. Screening (screening with cytology and/or HPV testing) 2. Colposcopy referral: Abnormal Pap test; hrHPV+; CC clinical suspicions and biopsy-confirmed CIN2+ | Population irrespective of HPV status | ✓ | ✓ |  |  |  |  |
| Zhang, 2017 | Colposcopy referral: HC2+ | hrHPV+ | ✓ |  |  |  |  |  |
| Yang, 2012 | Convenience sample: CIN1, CIN2, CIN3, CC | Population irrespective of HPV status |  | ✓ |  |  |  |  |
| Kong, 2020 | Convenience sample:   1. HPV16/18+ 2. ASCUS+ and HPV- 3. Negative control group (random sample): HPV- and cytology - | Population irrespective of HPV status | ✓ | ✓ |  |  |  |  |
| Agorastos, 2017 | Colposcopy referral: ASCUS+ or hrHPV DNA+ | 1. hrHPV+ 2. HPV16/18+ |  |  | ✓ |  | ✓ |  |
| Shi, 2017 | Convenience sample: ThinPrep cytology+ or HC2+ with suspected CIN | hrHPV+ |  |  | ✓ |  |  |  |
| Yu, 2018 | 1. Screening (screening with OncoE6, LBC, and Cobas HPV test) 2. Colposcopy referral: Abnormal Pap test; abnormal Pap test with hrHPV+; CC clinical suspicions and biopsy-confirmed CIN2+ | 1. hrHPV+ 2. HPV16/18+ |  |  | ✓ | ✓ | ✓ | ✓ |
| Rezhake, 2019 | Screening (screening with HC2) | hrHPV+ |  |  | ✓ | ✓ |  |  |
| Schweizer, 2010 | Convenience sample: negative histology, CIN1, CIN2, CIN3, CC | 1. Population irrespective of HPV status 2. HPV16/18+ |  |  |  |  |  | ✓ |
| Sellors, 2018 | Convenience sample: CIN1, CIN2, CIN3, CC; HPV+ and CIN- | HPV16/18+ |  |  |  |  | ✓ | ✓ |
| Torres-Ibarra, 2021 | Colposcopy referral: Cobas+ | HPV16/18+ |  |  |  |  | ✓ | ✓ |
| Zhang, 2017 | Colposcopy referral: VIA+ or LBC+ or HC2+ | hrHPV+ |  |  | ✓ | ✓ |  |  |
| Ndizeye, 2019 | WLWH attending the clinic | 1. Population irrespective of HPV status 2. hrHPV+ | ✓ | ✓ | ✓ | ✓ |  |  |
| Chibwesha, 2016 | WLWH attending the clinic | Population irrespective of HPV status | ✓ | ✓ |  |  |  |  |

Abbreviations: AGC= Atypical glandular cells; CC= Cervical Cancer; HC2= Hybrid capture 2; LBC= liquid-based cytology; VIA= Visual inspection with acetic acid

# **Description of all oncoprotein tests used in the 22 included studies**

**Supplementary table 5. Oncoprotein tests used and their technical principles**

| **Name, manufacturer** | **HPV types targeted** | **Technique and principle** |
| --- | --- | --- |
| **Oncoprotein test targeting the E6 protein** | | |
| OncoE6, Arbor vita (California, USA) – CE marked | 16/18 | - Qualitative test detecting elevated levels of E6 oncoproteins expressed by HPV 16 and 18 - Uses high-affinity monoclonal antibodies (mAb) in a lateral-flow assay format - Specific antibodies are immobilized on a nitrocellulose membrane where the analyte will migrate though capillarity - An alkaline phosphatase (AP) conjugated mAb will detect the analyte and the mAb-AP-analyte complex will be visualized by adding an enzyme substrate which will color the test line |
| OncoE6 Xiamen Bosheng Biotechnology Co., Ltd (Fujian, China) – Commercially available | 16/18 | - Qualitative test detecting E6 oncoproteins - Enzyme-linked immunosorbent assay (ELISA) - ELISA technique |
| In-house E6 test | 16/18 | 1. Yang et al. 2012 (Multicentric study Asia and America)  - Quantitative test detecting E6 oncoprotein - Antibodies against HPV E6 generated from mouse model - Non-HPV type-specific monoclonal antibodies obtained from mouse model - Whole-cell ELISA technique  1. Sellors et al. 2011 (Asia)  - Quantitative test detection E6 oncoprotein - E6 pull-down through PDZ interaction-mediated E6 precipitation - Western blot (10%/20% gradient criterion Tris-HCl polyacrylamide gel) |
| **Oncoprotein test targeting the E7 protein** | | |
| E7 protein assay, AMID Biotech (Tianjin, China) – Commercially available | 16/18 | - Qualitative test detecting the E7 oncoprotein - Magnetic particle–based chemiluminescence enzyme immunoassay - Double-antibody sandwich method |
| In-house E7 test | 16/18/45 | - Qualitative test detecting E7 oncoproteins - Sandwich ELISA assay - Combinations of rabbit monoclonal antibodies (RabMAbs) and polyclonal goat-anti-E7 antibodies |
| **Oncoprotein test targeting the E6 and E7 proteins** | | |
| 8-type HPV E6E7 oncoprotein testing, Arbor Vita (California, USA) – Newly developed, Research use only | 16/18/45/31/33/35/52/58 | - Qualitative test detecting elevated expression of E6 and E7 oncoprotein - Detection of E6 protein expressed by HPV types 16,18,31,35, and 45 - Detection of E7 protein expressed by HPV types 33,52, and 58 - Uses high-affinity monoclonal antibodies (mAb) in a lateral-flow assay format - Specific antibodies are immobilized on a nitrocellulose membrane where the analyte will migrate though capillarity - An alkaline phosphatase (AP) conjugated mAb will detect the analyte and the mAb-AP-analyte complex will be visualized by adding an enzyme substrate which will color the test line |
| OncoHealth protein test E6/E7, OncoHealth (San Jose, California) – Not commercially available anymore | Non type specific HPV E6/E7 AB | - Quantitative test detecting E6 and E7 oncoproteins - Direct E6/E7 HPV Whole-Cell ELISA - Detects non-type specific HPV E6 and E7 monoclonal antibodies |
|  |  |  |

# **Meta-regression analyses exploring the population (screening/triage vs colposcopy referral vs convenience sample) as covariate of interest**

**Supplementary figure 1: Accuracy of oncoprotein test for CIN3+ detection in the population irrespective of HPV status using the population as covariate**


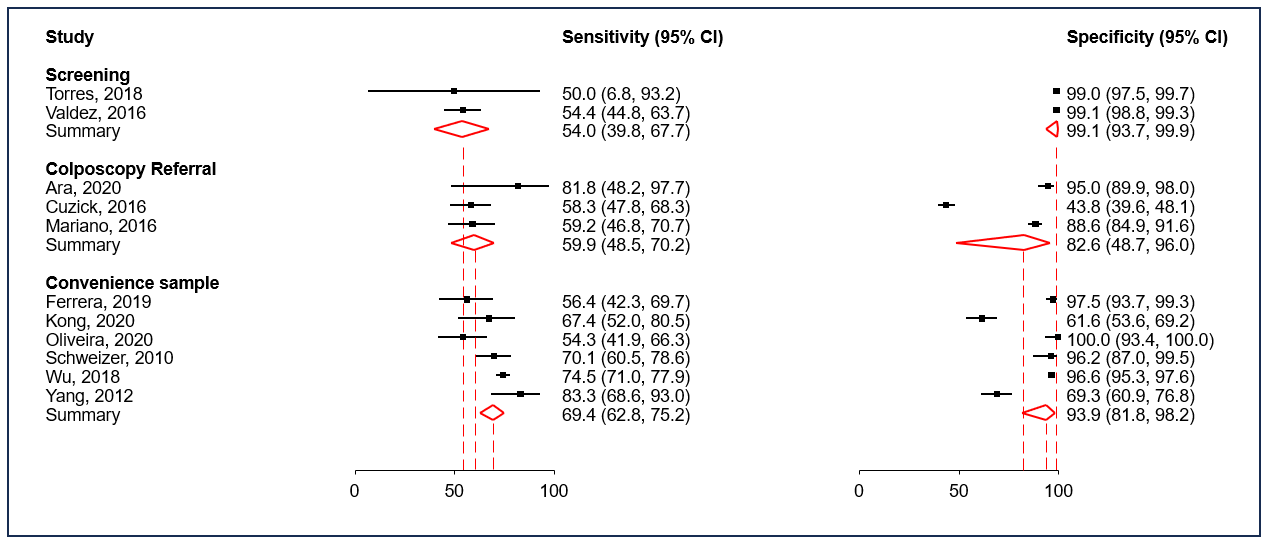


**Supplementary figure 2: Accuracy of oncoprotein test for CIN3+ detection in hrHPV+ women using the population as covariate**


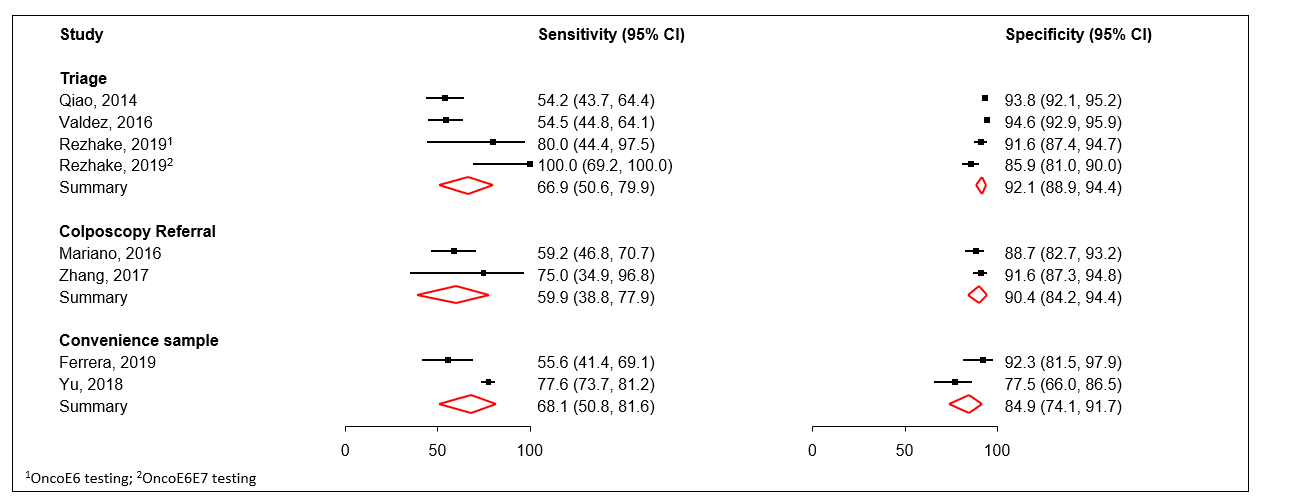


**Supplementary figure 3: Accuracy of oncoprotein test for CIN3+ detection in HPV16/18+ women using the population as covariate**


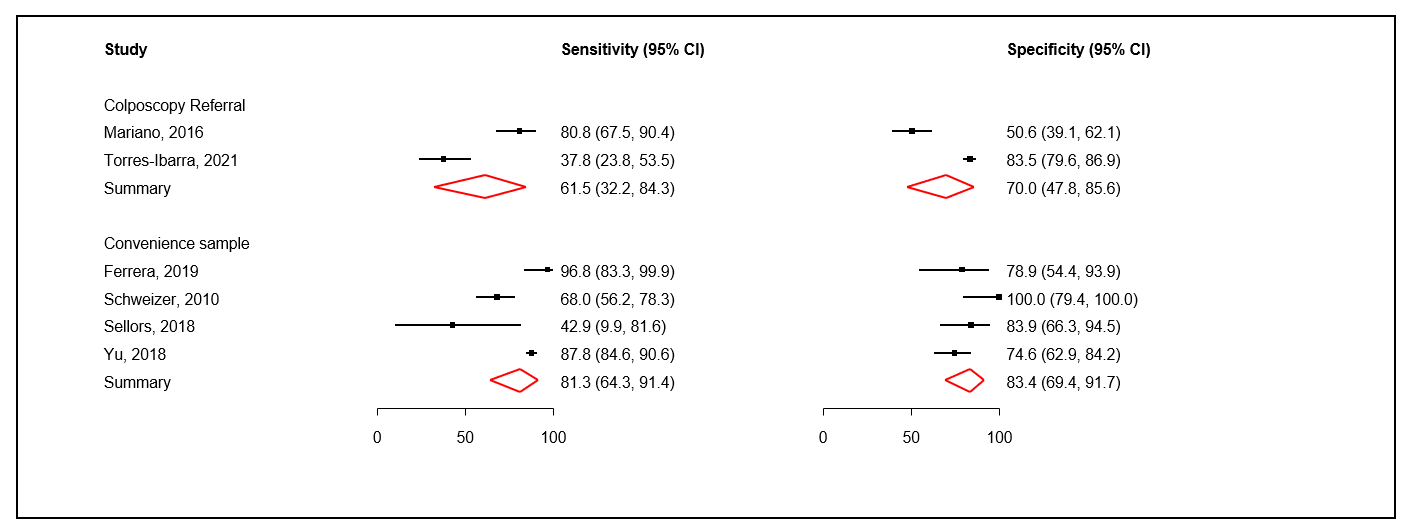


# **Accuracy of oncoprotein testing according to sample storage temperatures**

**Supplementary figure 4. Accuracy of oncoprotein test for CIN3+ detection by sample storage temperatures in the population irrespective of HPV status**


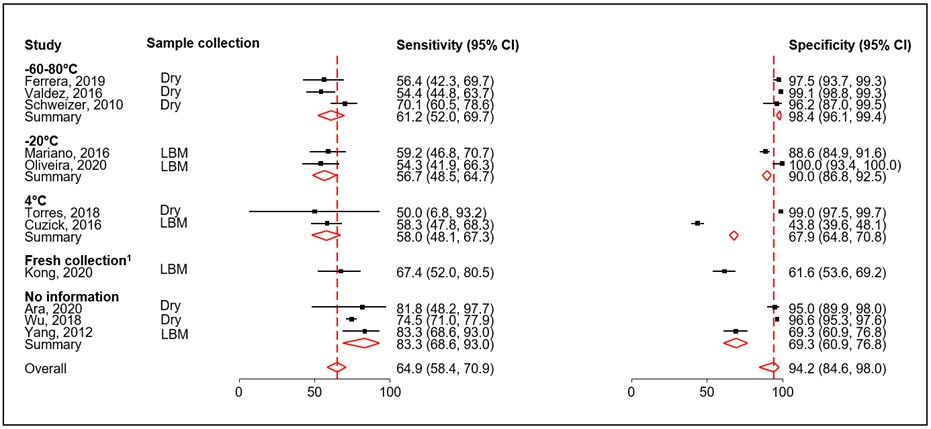


^1^Fresh collection usually defined as <24hours until test processing. Here, testing was done within 2 hours after collection. Abbreviation: LBM= Liquid-Based Medium

**Supplementary figure 5. Accuracy of oncoprotein test for CIN3+ detection in hrHPV+ women by sample storage temperatures**


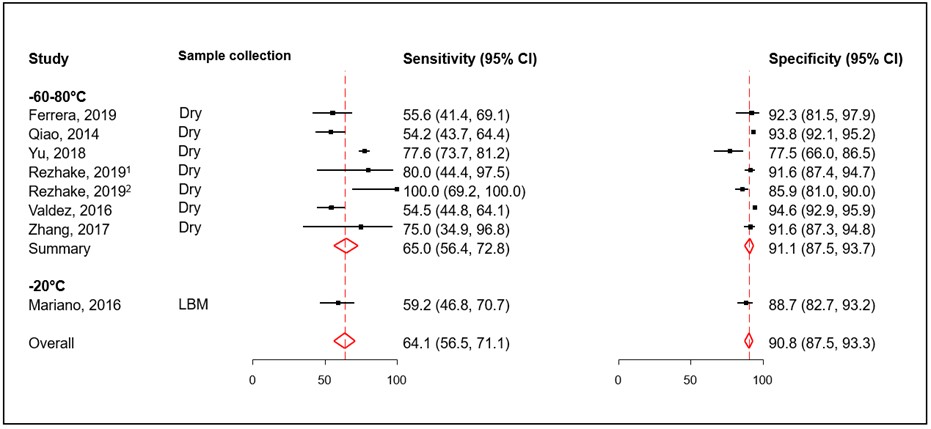


^1^OncoE6 testing; ^2^OncoE6E7 testing. Abbreviation: LBM= Liquid-Based Medium

**Supplementary figure 6. Accuracy of oncoprotein test for CIN3+ detection in HPV16/18+ women by sample storage temperatures**


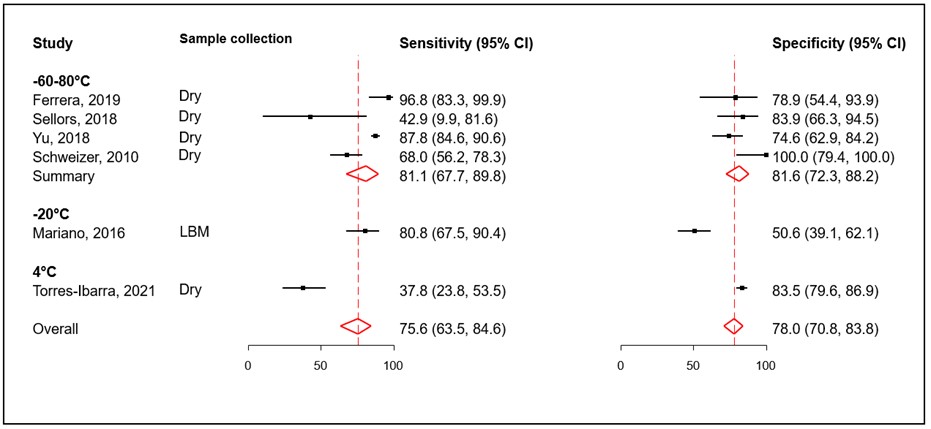


Abbreviation: LBM= Liquid-Based Medium

# **Accuracy of oncoprotein testing in studies with different country income levels**

**Supplementary figure 7. Accuracy of oncoprotein tests for CIN3+ detection by income level groups**


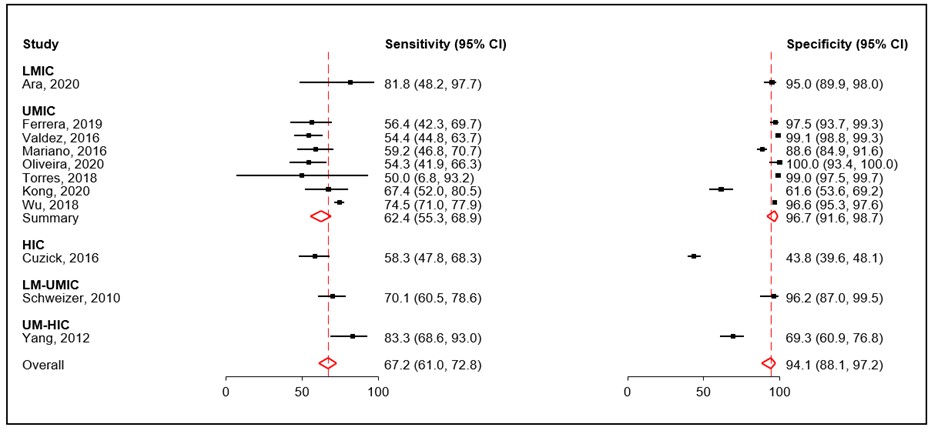


Legend: LMIC=Low-middle income country; UMIC= Upper-middle income country; HIC= High-income country; LM-UMIC= Low-middle – Upper-middle income country; UM-HIC= Upper-middle- High income country (World bank classification[1])

# **Accuracy of oncoprotein testing to detect CIN2+**

**Supplementary figure 8. Accuracy of oncoprotein tests for CIN2+ detection in the population irrespective of HPV status**


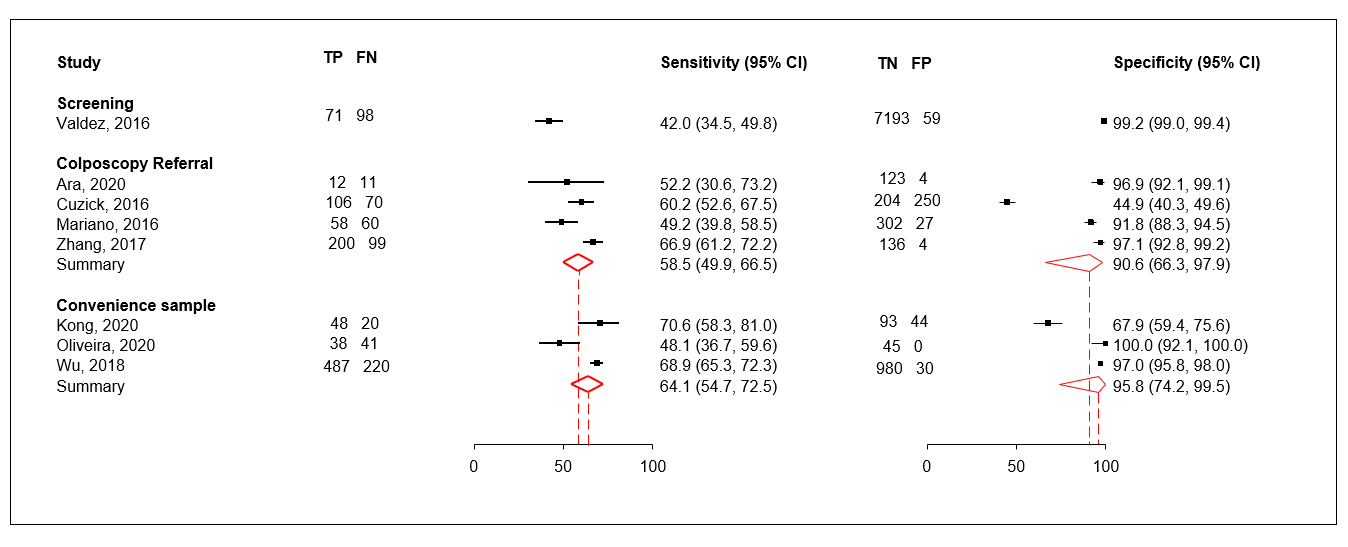


Legend: Results from a meta-regression using as a covariate the type of included population; Abbreviations: TP=True Positives, FN=False Negatives; TN=True Negatives, FP=False positives

**Supplementary figure 9. Accuracy of oncoprotein tests for CIN2+ detection in hrHPV+ women**


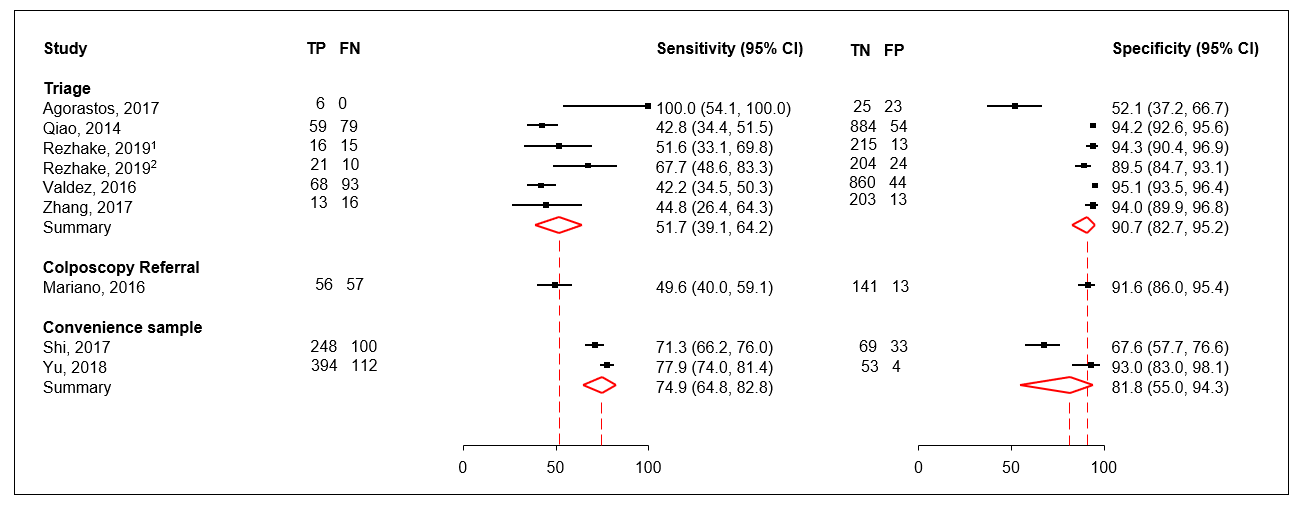


Legend: Results from a meta-regression using as a covariate the type of included population; ^1^Testing with OncoE6; ^2^Testing with OncoE6E7; Abbreviations: TP=True Positives, FN=False Negatives; TN=True Negatives, FP=False positives

**Supplementary figure 10. Accuracy of oncoprotein tests for CIN2+ detection in HPV16/18+ women**


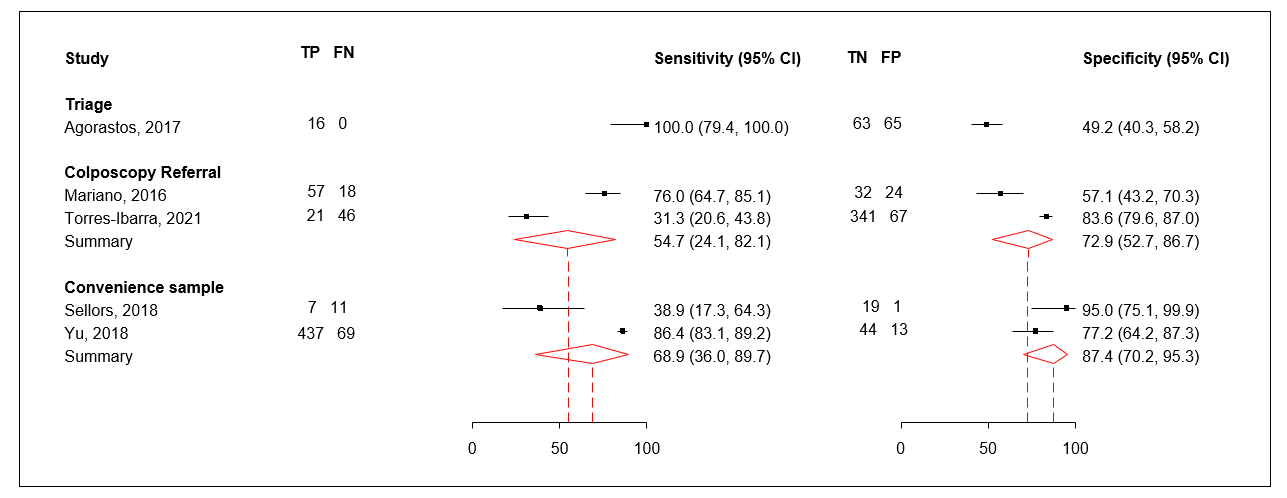


Legend: Results from a meta-regression using as a covariate the type of included population; Abbreviations: TP=True Positives, FN=False Negatives; TN=True Negatives, FP=False positives

**Supplementary figure 11. Accuracy of oncoprotein testing relative to VIA, cytology, HPV DNA/RNA testing and HPV 16/18 DNA testing for CIN2+ detection in the population irrespective of HPV status**

**
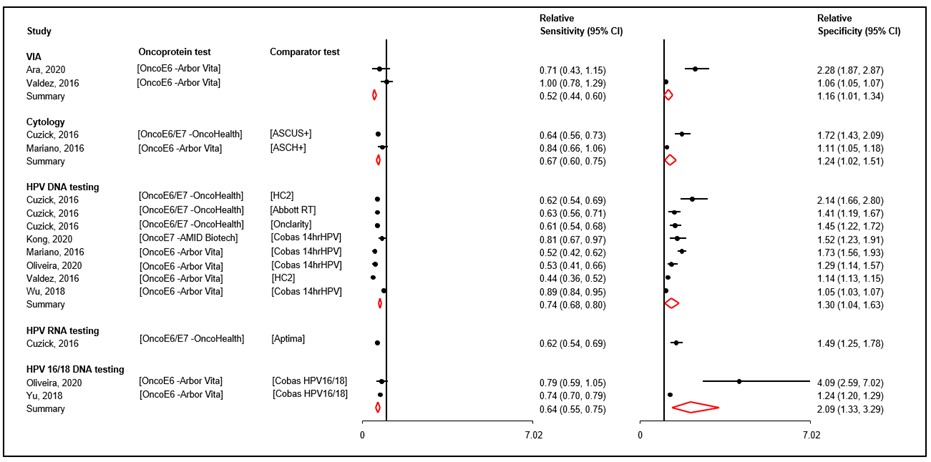
**

Legend: Results from a meta-regression using as a covariate the type of screening test. Abbreviation: ASCH+= Atypical Squamous Cells where High-grade squamous intra-epithelial lesions cannot be excluded; ASCUS+= Atypical Squamous cells of Undetermined Significance; HC2= Hybrid Capture 2; RT= Real Time

**
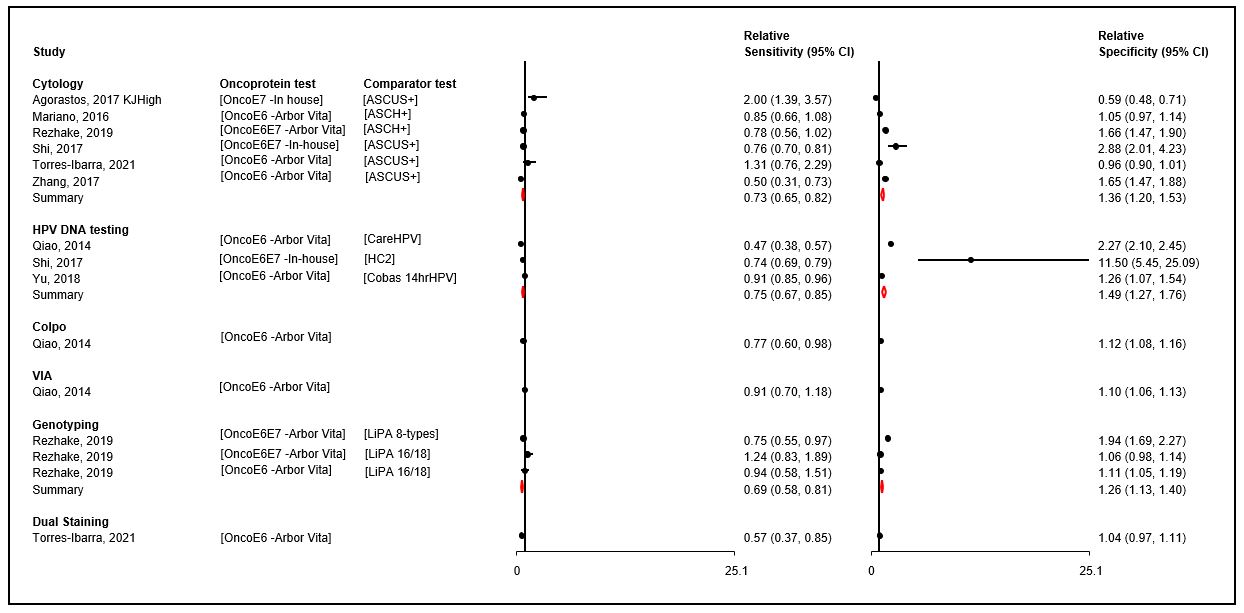
Supplementary figure 12. Relative accuracy of oncoprotein testing with HPV DNA and RNA testing, cytology, and VIA for CIN2+ detection in hrHPV+ women**

# **Quality assessment of the included studies reporting on the accuracy of oncoprotein testing**

**
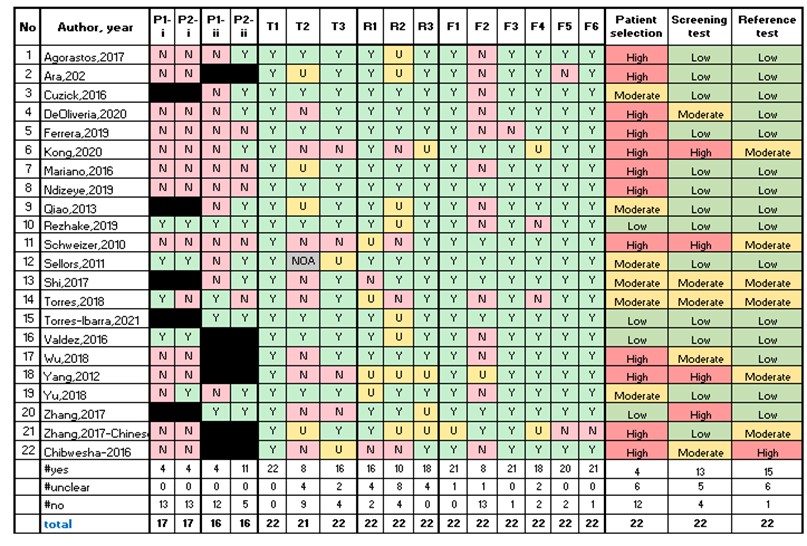
Supplementary figure 13. Assessment of the quality of diagnostic studies using the QUADAS-2* check list**

*****QUADAS= Quality Assessment of Diagnostic Accuracy Studies; NOA= Not of application

QUADAS items: (P1) acceptable enrolment method, (P2) inappropriate exclusions avoided, (T1) prespecified test cut-off, (T2) results of index and comparator tests blinded towards each other and toward reference test, (T3) results of index and comparator tests blinded towards gold standard, (R1) acceptable reference test, (R2) results of reference test blinded towards index and comparator tests, (R3) incorporation bias avoided, (F1) acceptable delay between triage tests and reference test, (F2) partial verification avoided, (F3) differential verification avoided, (F4) withdrawals explained, (F5) uninterpretable results reported for tests, (F6) uninterpretable results reported for reference test. Each quality item is judged with: Y (fulfilled, green), U (unclear, yellow), N (not fulfilled, red).

The QUADAS tool covers the risk of bias in four major domains that are patient selection, index test(s), reference standard, and flow and timing. In addition to risk of bias, the first free domains are also assessed for concerns regarding applicability. The number of studies with low, high, or unclear/moderate risk of bias regarding applicability for each domain were summarised in a graphical presentation. This quality assessment was performed by two independent reviewers (LD and IJ). A third reviewer (M. Arbyn) helped reaching consensus should any discrepancies arose between the two reviewers cited above.

**
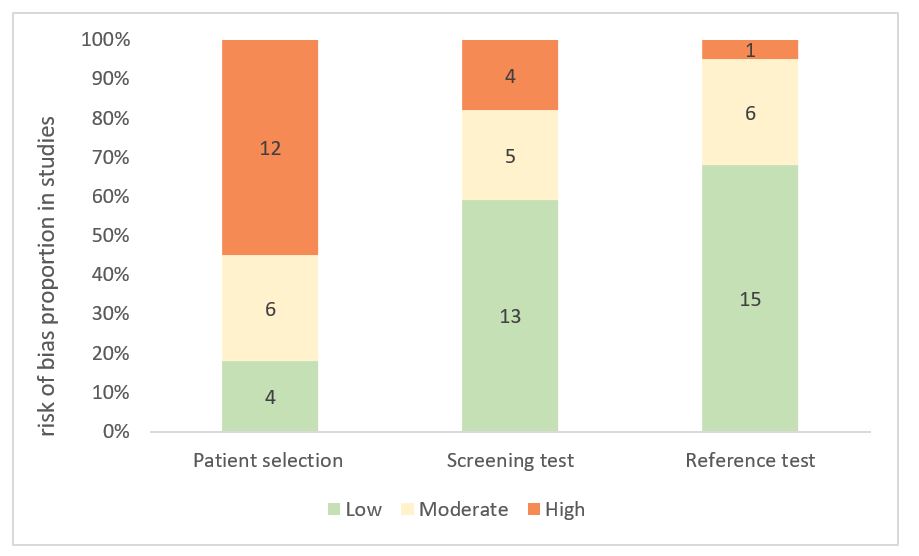
Supplementary figure 14. Risk of bias in the included studies using the QUADAS-2 tool**

Concerning the screening tests (index and others), the risk of bias was considered low in 59%, moderate in 23% and high in 18% of the 22 diagnostic studies. Information on the test positivity (e.g., targeted oncoproteins with corresponding HPV genotypes, cut-offs) and their execution (blinding of the oncoprotein test toward comparator and reference tests) was therefore predominantly good across all the included studies. Reporting on the quality of the reference test (valid reference, blinding toward comparator and reference tests, and avoiding incorporation bias) was of high, moderate, and low risk of bias in 68%, 27% and 5% of the studies, respectively. Almost all studies (95%) reported an appropriate time interval between the tests (index and comparators) and the reference standard of less than six months. None of the studies reported a delay between the tests of more than one year and less than 1% did not report on it. Although differential verification was avoided in 95% of the studies, partial verification was noted in 59% but absent in 36% of them. Reasons for withdrawing study subjects were clearly reported in 82%, but not reported or unclear in 9% of each. Most of the included studies did not report invalid results regarding the tests and the reference standard (91% and 95% respectively).

*Legend*: patient selection includes the following items from the QUADAS analysis “Representativity” and “Exclusion”; Screening tests includes the following items from the QUADAS analysis “Test positivity”, “Blinding screening test”, “Blinding tests vs gold standard”; Reference test includes the following QUADAS items “Valid reference”, “Reference blinded to tests”; “incorporation bias”.

**Supplementary figure 15. Accuracy of oncoprotein tests for CIN3+ detection by risk of bias in patient selection from QUADAS analysis**

**
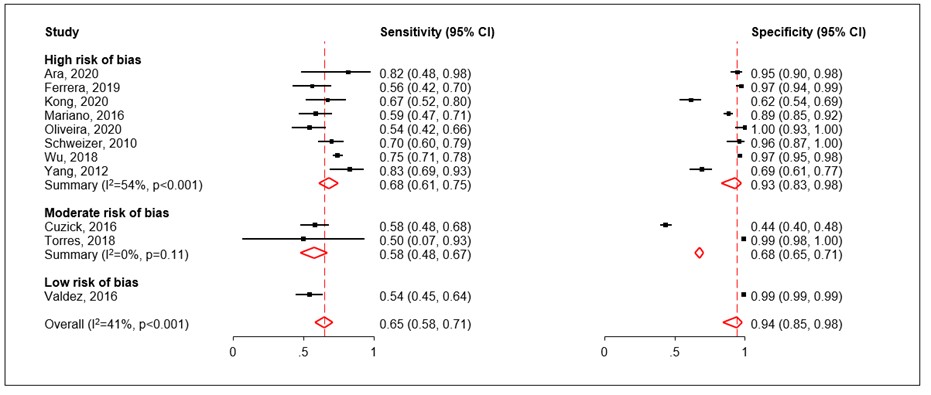
**Results from a stratified analysis by risk of bias in patient selection

**Supplementary figure 16. Accuracy of oncoprotein tests for CIN3+ detection by risk of bias in screening test from QUADAS analysis**


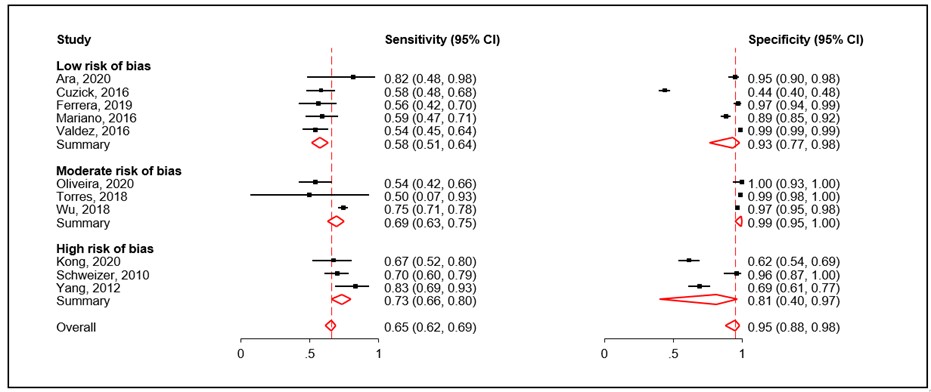


Results from a meta-regression using as a covariate score of risk of bias in screening test from QUADAS analysis (p-value for relative sensitivity moderate risk of bias vs low risk of bias p= 0.018; p-value for relative sensitivity high risk of bias vs low risk of bias p<0.001; p-value for relative specificity moderate risk of bias vs low risk of bias p= 0.20; p-value for relative specificity high risk of bias vs low risk of bias p=0.46)

**Supplementary figure 17. Accuracy of oncoprotein tests for CIN3+ detection by risk of bias in reference test from QUADAS analysis**

*
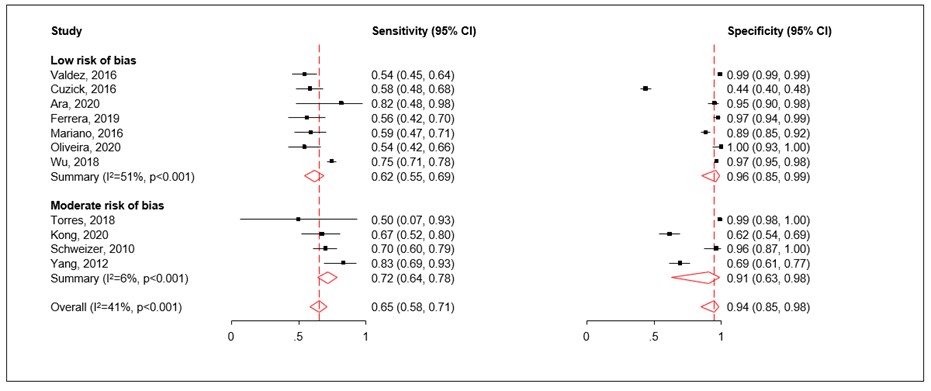
*

Results from a stratified analysis by risk of bias in reference test from QUADAS analysis

# **Specificity of oncoprotein testing as function of HPV prevalence**

**
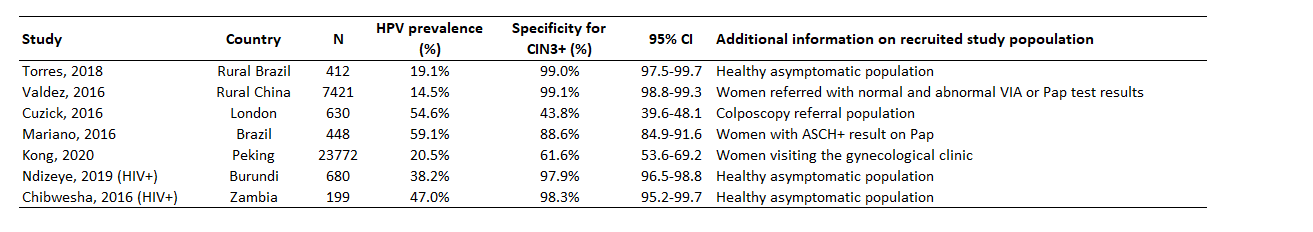
Supplementary table 6: Characteristics of the studies included in the meta-regression assessing the specificity of oncoprotein testing as function of HPV prevalence**

**Supplementary figure 18: Specificity of oncoprotein testing for CIN3+ detection as function of HPV prevalence**


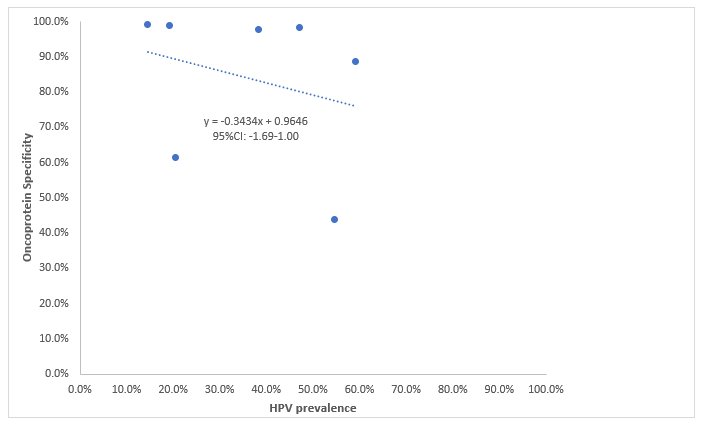


*Legend: meta-regression excluding studies using a convenience sample to avoid bias in true HPV prevalence*
